# Cellular reactions to long-term volatile organic compound (VOC) exposures

**DOI:** 10.1038/srep37842

**Published:** 2016-12-01

**Authors:** Johanna M. Gostner, Johannes Zeisler, Mohammad Tauqeer Alam, Peter Gruber, Dietmar Fuchs, Kathrin Becker, Kerstin Neubert, Markus Kleinhappl, Stefan Martini, Florian Überall

**Affiliations:** 1 Division of Medical Biochemistry, Biocenter, Medical University of Innsbruck, Austria; 2 Bioenergy 2020+, Graz, Austria; 3 Division of Biomedical Sciences, Warwick Medical School, University of Warwick, UK; 4 Division of Biological Chemistry, Biocenter, Medical University of Innsbruck, Austria; 5 ATLAS Biolabs GmbH, Berlin, Germany

## Abstract

Investigations of cellular processes initiated by volatile organic compounds (VOCs) are limited when modelling realistic long-term exposure scenarios at low concentrations. Exposure to indoor VOCs is associated with a range of adverse effects, but data on molecular changes at regulatory threshold limits are lacking. Activity analysis of VOC *in vitro* can be a valuable complement to inhalation toxicological evaluations. We developed an exposure platform that generates a stable VOC atmosphere and allows the exposure of cells for longer periods. Using formaldehyde as a model analyte, air-liquid interface cultured A549 lung epithelial cells were exposed to critical concentrations of 0.1 and 0.5 ppm for 3 days. Owing to the lack of known exposure biomarkers, we applied a genome-wide transcriptional analysis to investigate cellular responses at these sublethal concentrations. We demonstrate a minor overlap of differentially expressed transcripts for both treatment concentrations, which can be further analyzed for their use as exposure biomarkers. Moreover, distinct expression patterns emerge for 0.1 and 0.5 ppm formaldehyde exposure, which is reflected in significant enrichment of distinct biological processes. More specifically, metabolism of specific compound classes, lipid biosynthesis and lung-associated functions are affected by lower exposure levels and processes affecting proliferation and apoptosis dominate the higher exposure levels.

In recent years, special attention has been paid to volatile organic compounds (VOC) present in indoor environments and their impact through a variety of adverse effects^1^. Health effects associated with poor indoor air quality (IAQ) are most likely driven by chronic low-level exposure to some of these compounds, including gases such as ozone and carbon monoxide, but most importantly also to chemicals with high vapor pressure at room temperature, named VOCs^2^. VOCs originate from a variety of natural and anthropogenic sources and belong to different chemical classes, thus exhibiting distinct modes of action. Direct or acute toxicity is typically observed in only a few specific VOCs at concentrations occurring in indoor environments. More frequently observed effects include respiratory irritation, and certain VOCs have been suggested to be responsible for long-term health effects associated with the development of allergy and asthma^3^ and neuropsychoimmunological manifestations, such as “sick building syndrome”^4^.

Among these compounds, formaldehyde (HCHO) is of particular importance as it is a widely applied chemical. Formaldehyde toxicity can affect all organ systems in particular those which get into direct contact. The exerted effects range from carcinogenicity to irritation and allergy and depend from the concentration, the duration and the route of exposure. Owing to its hazardous potential, formaldehyde was included in the Community Rolling Action Plan for continuous evaluation.

Formaldehyde is readily soluble in water and is classified as a very volatile organic compound (vVOC)^5^. Formaldehyde gas is colorless, with a pungent, suffocating odor at room temperature. Olfactometrically determined thresholds for formaldehyde range from 0.04 to 0.4 ppm with some variation depending on the experimental setup and study groups^6^. Building materials and home furnishings are sources of indoor formaldehyde. Koeck *et al*. reported that approximately 20% of nearly 800 Austrian apartments reached concentrations above 0.1 ppm^7^, and concentrations in newly manufactured or mobile homes can be even higher^6^,^8^.

Exposure to high levels of airborne formaldehyde impairs lung function, though already lower levels can cause sensory irritation and favor asthma development^9^,^10^,^11^. Hence, minimal risk levels for airborne formaldehyde have been established, and the lowering of these exposure limits by legislation is the subject of ongoing debate. In the European Union, regulatory threshold limit values (TLV) are set at the national level. In Austria, 0.5 ppm is currently considered an occupational exposure level (OEL), and emission levels for building products are set at 0.1 ppm.

Our understanding of the effects of VOCs on human health is limited because of analytical difficulties in measuring real ambient air concentrations and in the evaluation of personal exposure as well as due to poor knowledge regarding the toxicity from multiple compounds^10^,^11^,^12^,^13^. In addition to the limitations of epidemiological studies, there is a substantial lack of basic molecular information on the mode of VOC action. Investigating the molecular effects of VOCs *in vitro* entails the resolution of two main challenges: (i) the need for a suitable exposure device that can generate a stable atmosphere continuously over a longer period and (ii) the knowledge gap about common key response genes or mechanisms perturbed at sublethal concentrations.

To test volatile compounds in the gas phase, a special exposure device that allows close contact between cultured cells and volatile substances with a precise control of gas concentration and without an interfering medium is necessary^14^. There are few systems available commercially^15^,^16^,^17^,^18^, also some groups assembled individual devices. Most systems are designed to be used with air-liquid interface (ALI) cultures of epithelial cells, where the test atmosphere is applied directly to the apical culture surface from the top of the exposure chambers. Thus, only low gas flow can be used, to avoid cell stress owing to increased gas pressure. Dehydration may occur as gas streams are not fully humidified, so exposure periods of only a few hours are recommended^18^,^19^,^20^. The almost exclusive usage of such systems may lead to a publication bias in the field of *in vitro* VOC activity assessment because short exposure time is a limiting factor when investigating effects at low level concentrations. Important mechanisms of adaptation and/or changes in metabolism that lateron trigger adverse effects are ignored.

The high humidity in the atmosphere is important for cell viability in prolonged exposures, but condensation must be avoided because absorption effects of the analyte in pipes, fittings and on other surfaces may become critical, in particular with low concentrations and stationary atmospheres or low flow rates. The exposure platform presented in this study provides humidification of the gas stream to ≥95% and a continuous high gas exchange rate and thus generates a stable atmosphere with a defined analyte concentration at optimal culture conditions. These conditions allowed the exposure of A549 cells at ALI to airborne formaldehyde at regulatory threshold concentrations over a period of 3 days.

A further critical aspect of *in vitro* testing is the choice of biological endpoints, in particular for exposure to low, sublethal concentrations, for which the determination of cell viability or oxidative stress induction is insufficient. In the cell, formaldehyde, which can also derive from endogenous processes, is rapidly catabolized to formic acid and further to carbon dioxide and water if not used in intermediate metabolism as one-carbon donor. However, exogenously added formaldehyde is suggested to non-specifically interact with proteins and macromolecules at the cell surface at concentrations before starting to penetrate into the cells, which hampers the selection of adequate biological endpoints. To investigate molecular changes in an unbiased manner, whole genome-based transcriptional analysis was used. Transcription is the first step in gene regulation, and is required for the dynamic adaptation of the cells’ proteome to different demands^21^.

To our knowledge, this is the first *in vitro* study on airborne formaldehyde exposure using a novel platform that considers such low concentrations and a continuous long-term exposure to identify associated signaling changes and potential biomarkers in a transcriptomics approach. This concept has the potential to be universally applied to obtain insight into VOC-induced molecular changes that occur at sublethal concentrations, and is thus of relevance to unravel the molecular basis of potentially harmful effects at low-dose exposures.

## Results

### Development of an exposure platform

**Figure 1:**
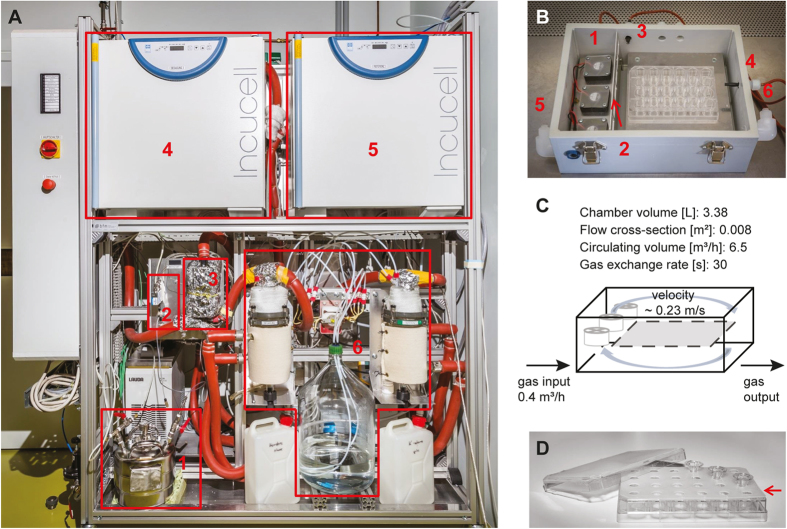
Exposure incubator platform. (**A)** The system consists of an exposure setup, where liquid compounds (1) are injected via a mass flow controller (2) into the heating system (3). Vaporized compounds are continuously fed into a carrier gas line, which feeds into a transfer line, directing the gas mixture to the exposure incubator (4). A reference incubator is used for control measurements with independent transfer gas only (5). All gas streams are humidified (6, humidification unit) and reach cell cultures at 37 °C and ≥95%_rel_ humidity. **(B)** Cell culture plates are deposited into sterilized lockable chambers, where fans (1) for internal fast circulation and lattices (2) ensure a homogeneous distribution. These boxes contain two temperature sensors (3, 4) that provide information on the surrounding climate; the boxes are connected to the gas supply with inlet- (5) and outlet-(6) fittings. **(C)** Theoretical estimation of flow proportions in the chambers. **(D)** A silicon pad holder blocks carryover of VOCs into the medium without passing the cell layer. Lids covered with fine-mesh nettings were used instead of standard culture plate lids.

**Figure 2:**
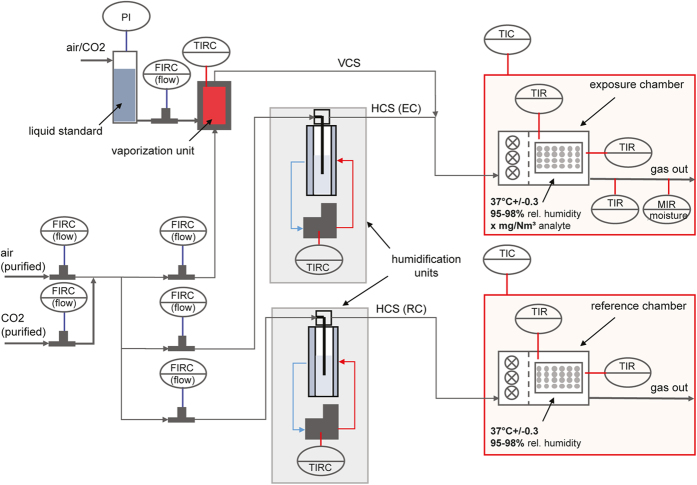
Process control interface. Schematic presentation of the process design showing the liquid and gas flows, the humidification units, and the exposure and reference chamber (VCS: vaporized carrier stream, HCS: humidified carrier stream, EC: exposure chamber, RC: reference chamber, FIRC: flow/indicated/registered/controlled, MIR: moisture/indicated//registered, TIC: temperature/indicated/controlled; TIR: temperature/indicated/registered, TIRC: temperature/indicated/registered/controlled, PI: pressure/indicated).

The exposure platform (Fig. 1A) was designed to enable optimal treatment of cells with a humidified atmosphere containing a volatile analyte, formaldehyde, in a defined concentration. Exposure was realized by using a liquid formaldehyde standard that was injected via a liquid mass flow controller (LMFC, accuracy 1% of full scale value [%_FS_]) into a heating system, undergoing complete evaporation (Fig. 2). The evaporated analyte was transferred by a carrier gas stream (vaporized carrier stream, VCS) and mixed with a humidified carrier gas stream (HCS). The dry gas was saturated in a water bubbling system with a defined temperature. Both gas streams consisted of purified, dry air and 5% CO_2_. The accuracy of gas mass flow controllers (MFC) was +/−1%_FS_. Conditions in the exposure chamber were 37 °C (+/−0.1 to 0.5 °C), 95 to 98% relative humidity, and a defined analyte concentration. The exposure chamber was equipped with temperature sensors at two positions within the chamber and a humidity sensor at the gas outlet (Fig. 1B).

To guarantee a homogenous distribution of the analyte gas, three fans were installed in the chamber (Fig. 1B and C ). The analyte gas flow was 400 L/h, and the circulating volume was approximately 6,500 L/h because of the fans. The exhaust gas flowed into the exhaust air system of the laboratory. A second chamber was installed as a reference and equipped with only temperature sensors. The gas supply for this chamber was installed as above, but without the analyte-carrying gas stream.

The humidity in the chambers is a critical parameter for experiments of long duration, because the continuous gas flow can dry off considerable quantities of fluid, reducing cell viability. A deviation of 1 °C from the optimal chamber temperature of 37 °C would lead to an approximate decrease of 5% in relative humidity, assuming a theoretical relative humidity of 100% at 37 °C. Although the system has a continuous gas exchange rate, humidity ≥95% results in fluid losses comparable to the medium loss in a standard cell culture incubator as compared over the 3 day incubation period.

The exposure system was operated with a process control software. Adjustable experimental parameters included the concentration of the liquid standard, the gas flows, the heating systems, and the temperature of the humidification unit. Although an automated regulation of humidity was possible, the humidity was adjusted manually to avoid fluctuations and condensation. All process data were stored automatically. After each experiment, all critical parameters (temperature, humidity, flow) were controlled to assure a constant quality over the entire experimental time (Supplemental file S1).

### Exposure of cells to liquid and airborne formaldehyde

When added into medium to submerged A549 cells for 24 to 72 h, half maximal inhibitory concentration (IC_50_) values for formaldehyde were 1.390 mM (95% lower and upper limits: 1.198 mM; 1.620 mM) for 24 h, 0.850 mM (0.676 mM; 1.068 mM) for 48 h and 0.700 mM (0.568 mM; 0.863 mM) for 72 h of treatment.

**Figure 3:**
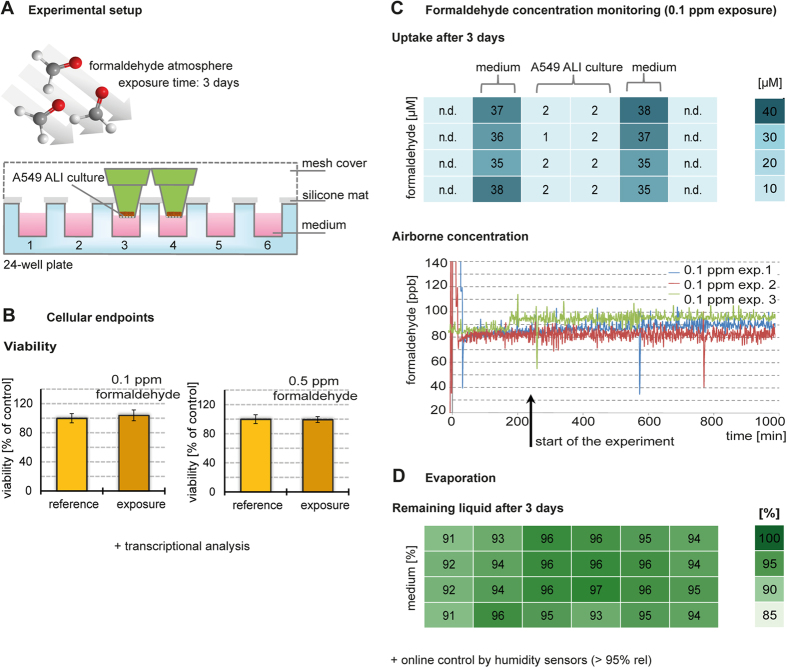
Schematic illustration of the exposure experiments. (**A)** ALI cultures of A549 cells were placed in the center wells of a 24-well plate and exposed to formaldehyde. **(B)** After 3 days, the viability of ALI A549 cultures exposed to gaseous formaldehyde at 0.1 ppm (and 0.5 ppm) was determined in comparison to cells cultivated in the reference chamber. Mean values ± SEM of 3 experiments are presented. Cells were harvested and RNA was extracted for transcriptional analysis. **(C)** The uptake of formaldehyde, which dissolved in the medium, was measured by HPLC after day 3 from wells in rows 2 to 4. Mean concentrations of 3 experiments with 0.1 ppm formaldehyde are presented in the 24-well scheme (n.d., not determined). Formaldehyde concentration in the headspace of the exposure chamber was measured at 1-minute intervals for 3 different experiments (set parameters of exposure platform: 0.1 ppm formaldehyde). **(D)** The evaporation profile was determined by weighing the remaining medium in each well after 3 days. Humidity in the gas phase was continuously monitored by sensors (Supplemental file 1).

For the airborne exposure experiments, A549 cells were grown to confluence and lifted to ALI. After 24 h, A549 ALI cultures were placed into fresh medium, and a silicon mat was used as a seal to avoid any direct gas entry into the medium. ALI cultures were placed in the middle rows of 24-well plates; wells at the edges were filled with medium only (Fig. 3A). Treatment conditions were adjusted at the exposure platform at least 4 hr before the start of the experiments so that temperature, humidity and analyte concentration were balanced until the cells were placed into the exposure or reference chamber. ALI cultures were exposed to atmospheres containing 0, 0.1, and 0.5 ppm formaldehyde for 3 days. Experiments were performed in triplicated in a randomized order.

Cell viability was estimated as a primary endpoint after exposure, using a metabolic activity assay based on the reduction of resazurin. For both treatment concentrations, cultures from the exposure chambers showed no differences in viability compared with cultures from the reference chambers (Fig. 3B).

Formaldehyde is highly water-soluble, so the uptake in the culture medium could be measured after 2,4-dinitrophenylhydrazine (DNPH) derivatization with HPLC analysis (Fig. 3C). After 3 days, mean formaldehyde uptake in wells filled with medium only was 36.40 ± 2.07 μM with the 0.1 ppm atmosphere and 206.44 ± 12.87 μM with the 0.5 ppm atmosphere (Table 1). Airborne formaldehyde did not significantly accumulate in the medium below the ALI cultures (e.g. mean of 4.06 ± 1.86 μM with the 0.5 ppm atmosphere), as measured concentrations were comparable to background levels (mean of DNPH blank: 3.26 ± 0.77 μM formaldehyde). The limits of detection (LOD) and of quantitation (LOQ) were calculated based on determinations of blank samples and were 5.56 μM (LOD) and 10.61 μM (LOQ), respectively. In addition, no formaldehyde could be detected in sporadic control measurements of the medium below the cells in the reference chamber (4.37 ± 0.17 μM). Thus, A549 culture surfaces were exposed to substantial amounts of formaldehyde.

For a number of experiments, the amount of formaldehyde in the headspace of the exposure chambers was measured over a longer period by using the AL4021 formaldehyde analyser (Aero-Laser GmbH, Germany). The detection of formaldehyde by the AL4021 analyser is based on the Hantzsch reaction using acetylacetone as derivatization reagent. With the AL4021 analyser the reliability of the dosing and exposure could be further confirmed, as the concentrations at the platform´s setpoints were measured. A representative diagram comparing a calibration measurement and with a 0.5 ppm exposure can be found in supplemental file S1 (S1-Fig. 10A). In addition, interferences from formaldehyde deriving from potential air contamination in the carrier gas stream could be excluded, as only traces of formaldehyde were detected (approximately 4.5 ppb, see fig. S1-Fig. 10B in supplemental file S1). From the headspace of the exposure chamber, a minor part of the gas phase (60 L/h) was transferred into a separate heated gas line for analysis, and concentration was measured at 1 minute intervals continuously over a period of 16 h. Figure 3C shows the estimated concentrations for the 3 exposures to 0.1 ppm formaldehyde (uncorrected graphs). As the gas stream was humidified to greater than 95%_rel_, the additional water intake in the absorber solution leads to fluctuations and the minor deviation from the set concentration.

Loss of culture medium over longer exposure times could not be fully avoided because of the gas exchange rate, therefore evaporation of medium was controlled by weighing the remaining medium after 3 days of culturing (Fig. 3D). The quantity of medium lost depends on the gas exchange rate and the relative humidity of the gas stream, and thus on the accuracy of chamber and gas stream temperature maintenance. Experimental parameters were set to ≥95%_rel_ humidity, which accounts for a theoretically maximal allowed temperature difference (∆T_below saturation_) of 1.0 °C. After 3 days of culturing, approximately 90% of the initial medium volume remained (Table 1). For 24-well plates, where each well was initially filled with 2.6 mL, the highest total fluid loss was approximately 11% (6.9 mL). The maximum theoretical capacity of the total 95%_rel_ gas flow to 100%_rel_ humidity over 3 days would have been approximately 85 mL. Thus, the selected settings prevented excessive dehydration and assured optimal culture conditions during exposure. Data for medium evaporation and formaldehyde intake for each individual experiment are provided in supplemental file S1.

### Identification of candidate biomarkers

To explore cellular changes of A549 ALI cultures exposed to sublethal concentrations of formaldehyde, we performed a transcriptional analysis. For each treatment concentration, a whole genome expression array (GeneChip^®^ Human Genome U133 Plus 2.0, Affymetrix, Santa Clara, CA, USA) experiment was performed (GEO accession number: GSE76790).

**Figure 4:**
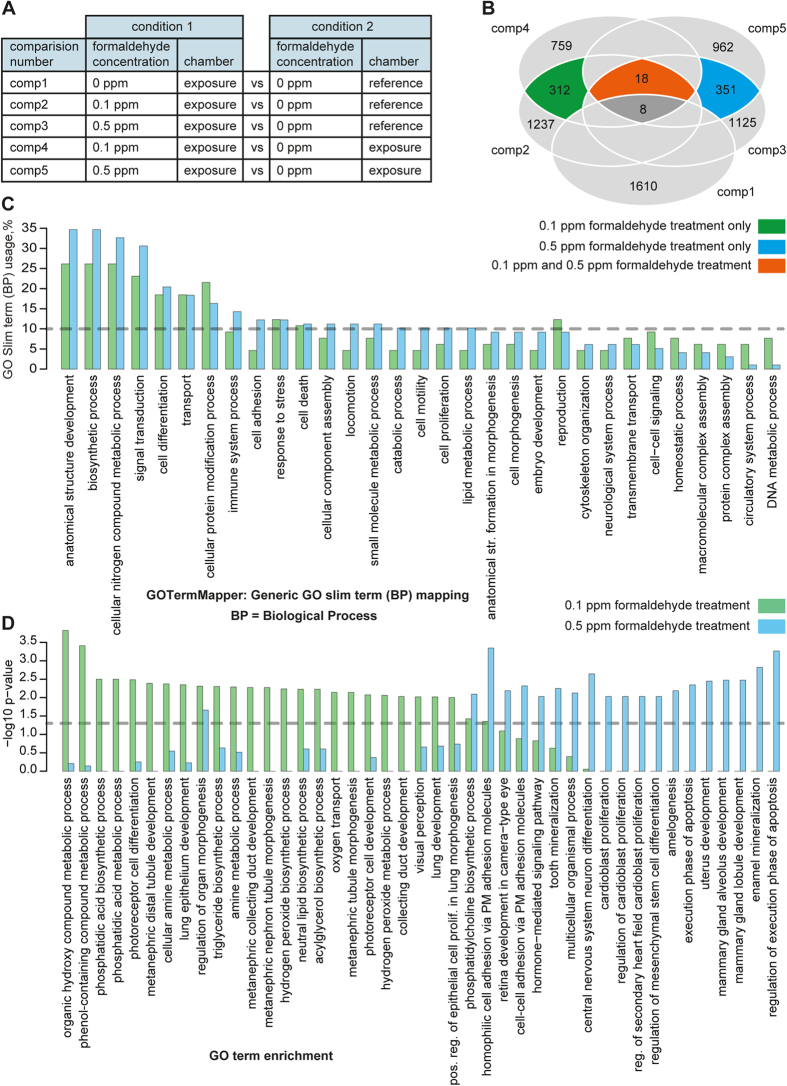
Analysis of differentially expressed transcripts due to formaldehyde exposure. (**A**) Overview of the comparative analysis of distinct treatments to obtain the differentially expressed gene sets. Comparisons 1 to 3 (comp1, comp2 and comp3) refer to the cells that were contemporaneously cultivated in the reference chamber. In comparisons 4 and 5 (comp4, comp5), the samples exposed to 0 ppm formaldehyde in the exposure chamber were used as control to exclude random background fluctuations due to chamber and time effect. (**B**) Venn diagram of the overlap of differentially expressed genes (p value <0.05) in each comparison. Numbers in the red compartment are genes that are regulated with both treatment concentrations, those in the green compartment are specific for 0.1 ppm, and numbers in the blue compartment are for 0.5 ppm formaldehyde exposure (detailed figure is provided in supplementary file S2). (**C**) To assess biological function, the differentially expressed transcripts were mapped to generic GO slim terms (http://go.princeton.edu/cgi-bin/GOTermMapper). Percentage usage of differentially expressed transcripts owing to 0.1 and 0.5 ppm exposure including the 18 common transcripts in generic GO terms are shown in light green and light blue bars, respectively. (**D**) Enrichment of GO biological processes owing to 0.1 and 0.5 ppm formaldehyde exposure. Only significantly enriched processes (*p*-value <0.05) in either concentration exposure are presented in the bar plot. Light green and light blue bars represent enrichment of differentially expressed transcripts owing to 0.1 and 0.5 ppm formaldehyde exposure, respectively. Grey dotted line marks the significant enrichment of biological process (*p*-value <0.05). (pos. reg. of epithelial cell prolif. in lung morphogenesis = positive regulation of epithelial cell proliferation involved in lung morphogenesis”; PM = plasma membrane; reg. = regulation).

Each experiment was performed in triplicates. The chronological order of experiments was randomized. Formaldehyde concentration was changed in the atmosphere of the exposure chamber from 0 to 0.1 and 0.5 ppm; no formaldehyde was present in the reference chamber (=control). Differentially expressed transcripts were determined for each parallel treatment including the control experiment with 0 ppm in the exposure chamber, which was performed to assess potential influences of the two different incubators (comp1, comp2 and comp3 in Fig. 4A). To take into account an influence of time of experiment, two additional comparisons were performed to identify the differentially expressed gene upon formaldehyde exposures (comp4 and comp5 in Fig. 4A). Therefore, 0.1 ppm and 0.5 ppm samples were compared with the contemporaneously cultivated samples in the reference chamber and the 0 ppm exposed samples in the exposure incubator to remove potential background effects^22^. Transcripts were considered differentially expressed owing to 0.1 ppm formaldehyde treatment when they are differentially expressed in comp2 and comp4 and not in comp1. Similarly, when a transcript is differentially expressed in comp3 and comp5 and not in comp1 then it is considered differentially expressed owing to 0.5 ppm treatment of formaldehyde. Overlapping sets of transcripts from these comparisons were used for further detailed analysis.

**Figure 5:**
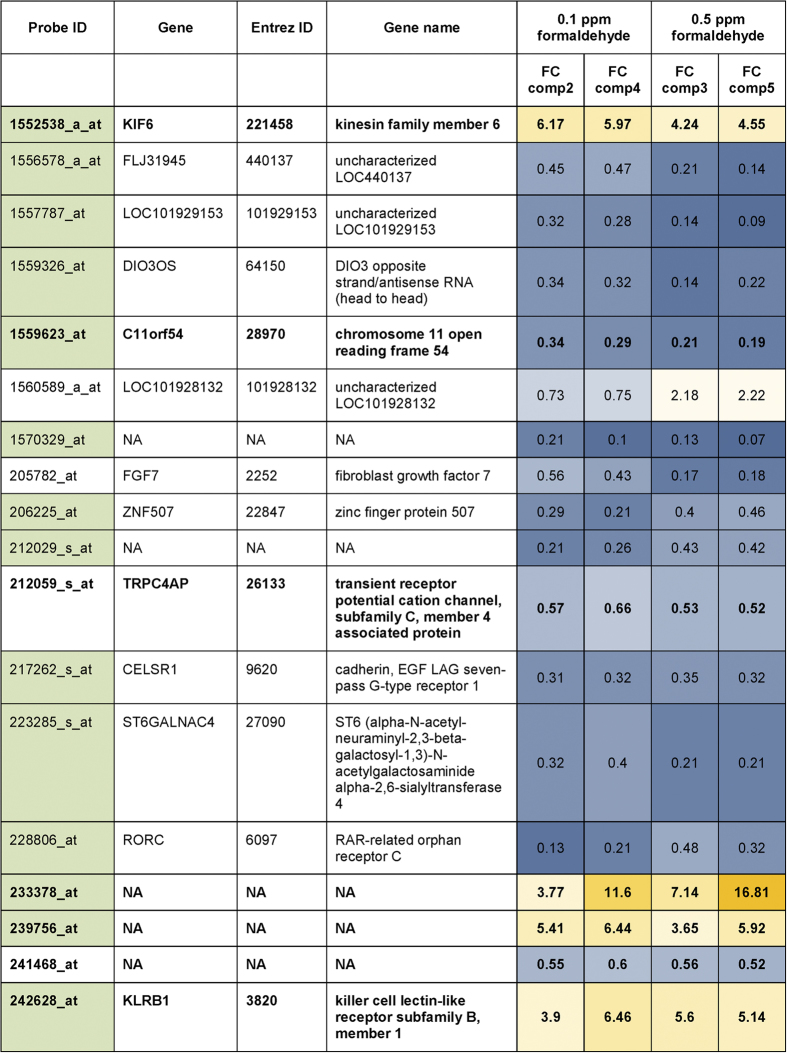
List of the common transcripts that are differentially regulated upon 0.1 and 0.5 ppm formaldehyde exposure in A549 cells, calculated without using fold change limitations. Probesets in green are those transcripts that are common when using fold-change threshold of 2 (FC = |2|) and a *p*-value cutoff of ≤0.05. Mean FCs are labelled in blue for downregulation and in yellow for upregulation compared to the respective controls. Probesets in bold indicate those transcript hits that were found also in the Anova/Tukey’s HSD approach.

With these comparisons potential influences of chamber and time were considered as random fluctuations of gene expression, and the resulting transcript sets represent those genes that are specifically regulated owing to formaldehyde exposure with 0.1 and/or 0.5 ppm, as indicated in the Venn diagram in Fig. 4B (more detailed in Supplemental file S2, S2-Fig. 1). Exposure of cells to 0.1 ppm resulted in 312 specifically differentially expressed transcripts, and 351 transcripts were specifically differentially expressed owing to 0.5 ppm formaldehyde treatment (*p-*value <0.05). Only 18 transcripts were affected with both treatment concentrations (Fig. 5). An extension of this analysis using a more stringent cutoff for differential expression (fold-change threshold of 2 and a *p*-value cutoff of <0.05) resulted in 138 transcripts specific for 0.1 formaldehyde exposure, 166 transcripts specific for 0.5 formaldehyde exposure and 14 common expressed transcripts. Detailed information and transcript lists can be found in Supplemental file S2 and Supplementary dataset file S3.

The analysis of the differentially expressed transcripts revealed only a minor overlap of regulated genes with both concentrations, and each exposure concentration resulted in an individual expression profile. To assess the roles of differentially expressed transcripts owing to 0.1 and 0.5 formaldehyde treatments separately in biological processes, they were mapped to generic GO slim terms (http://go.princeton.edu/cgi-bin/GOTermMapper) (Fig. 4C). Despite the differences at transcript level for 0.1 and 0.5 formaldehyde exposure, they largely participate in similar generic GO terms. Approximately one third of differentially expressed transcripts were mapped to generic GO terms, however, a large number of genes were not annotated and remained uncategorized (65 out of 222 (29.3%) for 0.1 ppm formaldehyde exposure, and 98 out of 266 (36.8%) for 0.5 ppm exposure). Biological processes with the highest assignment (≥15% genes usage) include cellular nitrogen compound metabolism, signal transduction, and biosynthetic processes, functions associated with the modification of cellular proteins, anatomical structure developments, transport and differentiation. Processes affecting the immune system, cell death and stress response were among those functions with an assignment of ≥10% genes usage. The strong difference in 0.1 and 0.5 ppm formaldehyde exposure at the transcript level depicted by the significant enrichment of different GO biological processes, where certain pathways are predominantly covered by one of the two treatments (Fig. 4D). Treatment with 0.1 ppm formaldehyde mainly affect metabolic processes of organic hydroxy compounds, phenol-containing compounds, cellular amines, hydrogen peroxide, and in particular of lipids. In addition, lung specific processes such as epithelium development and oxygen transport are targeted. Exposure to 0.5 ppm formaldehyde significantly targets specific processes involved in cell adhesion, differentiation and proliferation that have been characterized largely in other organ and cell systems such as the central nervous system, cardioblasts or the mammary gland. Biological processes that are significantly affected by both concentrations of formaldehyde (*p*-value <0.05) include phosphatidylcholine biosynthesis, homophilic cell adhesion via plasma membrane adhesion molecules and regulation of organ morphogenesis. Furthermore, we mapped the differentially expressed genes for each concentration (0.01 and 0.5 ppm formaldehyde) to other Gene Ontology Aspects such as Function and Component. As far as GO Function is concerned, ion binding function (GO:0043167) mapped with the maximum number of differentially expressed genes in each cases (11 out of 66 in the 0.1 ppm exposure, and 27 out of 98 in the 0.5 ppm exposure), followed by DNA binding (GO:0003677), nucleic acid binding transcription factor activity (GO:0001071), signal transducer activity ( GO:0004871) to name a few (full list can be found in supplemental file S3). In terms of GO Components, the differentially expressed genes are mainly cytoplasmic and plasma membrane genes, and very few are mitochondrial (full list is contained in supplemental file S3). It is important to note, however, that despite different genes are differentially expressed in each concentrations leading to enrichment of different Biological Processes (Fig. 4D), they mapped to similar Components and Biological Functions.

Out of the 18 probesets that were common for 0.1 and 0.5 ppm exposures (Fig. 5), 13 transcripts were mapped to genes. When comparing the average fold changes, only one transcript was downregulated with the lower concentration but upregulated in response to higher formaldehyde concentrations, whereas the direction of regulation was the same for both treatment concentrations for the remaining transcripts. Owing to the use of low exposure concentrations it can be expected that changes are near the detection limit. In addition to the eBayes approach, which uses a modified t-statistics that is advantageous for a small number of biological replicates, we applied analysis of variance (ANOVA) followed by Tukey’s HSD post-hoc test to consider the variance of the different controls (0 ppm exposure control and reference chamber control). It confirmed 7 of the 18 probesets that were identified by the eBayes approach. More detailed results from both methods are available in Supplemental file S2 (S2-Fig. 2), where also the expression of the common probesets is displayed in the heatmaps separately for the eBayes and ANOVA approaches.

## Discussion

This *in vitro* approach confirms the feasibility of investigating cellular responses to VOCs in a long-term airborne formaldehyde exposure scenario. This approach can be applied to a broad range of chemicals in the future and contribute to safety assessment by unravelling responses that may favour adverse outcomes.

A549, a human pulmonary epithelial cell line derived from a lung carcinoma^23^ was used. This cells are widely used as a cell model for toxicity screening^24^,^25^, as submerged cultures and ALI^26^, despite their relatively active antioxidant machinery^27^,^28^. They maintain the properties of phenotype alveolar epithelial (AE) type II cells^29^. Although AE type II cells compose only a small part of the alveolar surface, they constitute ~60% of overall alveolar epithelial cells and are responsible for synthesis, secretion and reuptake of surfactant, metabolism of xenobiotics, synthesis and secretion of immunomodulatory proteins, transepithelial movement of fluids and regeneration of the alveolar epithelium^30^,^31^,^32^. Thus, AE type II cells are not only important for the functional capabilities of the pulmonary system but also for mediation of the response to toxic compounds that may cause lung damage^30^,^33^.

To realize the exposure scenario, an incubator platform was designed that generated a stable formaldehyde atmosphere in a humidified environment. In this system, cell cultures were exposed to the gaseous analyte in a continuous manner; a relatively fast gas exchange rate guaranteed the presence of formaldehyde at selected concentrations over the whole incubation period. This fast gas flow without decreasing cell viability was only possible due to the tight control of humidity and temperature and had the advantage that any potential adsorption/desorption effects can be excluded.

The results from both online measurements and accumulated formaldehyde concentrations in control wells confirmed that A549 ALI culture surfaces were exposed to substantial quantities of airborne formaldehyde. Exposure to 0.1 ppm and 0.5 ppm led to an uptake of formaldehyde in medium in the μM range (approximately 35 and 200 μM), which did not affect viability. Of note, airborne formaldehyde did not efficiently accumulate in the medium below the A549 ALI cell layers. This finding is consistent with reports from *in vivo* studies where airborne exposures did not additionally increase the levels of naturally occurring formaldehyde in human blood^34^,^35^. When meeting the culture surfaces, formaldehyde reacts e.g. with amino groups of mucosal molecules. In the presence of water formaldehyde becomes nearly completely hydrated, thereby forming methylene glycol^36^.

Besides the reaction with cell surface molecules, cellular metabolism may contribute to formaldehyde clearance. A recent study of Pidoux *et al*. showed in a placental perfusion experiment with ^14^C-labelled formaldehyde that formaldehyde added at a concentration of 100 μM was able to cross and accumulate in the human placenta and interfered with cellular functions, thus exerting endocrine toxicity^37^. In the cell, formaldehyde reacts with reduced glutathione to form S-(hydroxymethyl)glutathione, which is then converted to S-formylglutathione via a class III alcoholdehydrogenase (ADH5), and further to formate by S-formylglutathione hydrolase (ESD)^38^,^39^. Formaldehyde oxidation is also possible via the mitochondrial aldehyde dehydrogenase (ALDH2). No significant regulation on gene expression level of the formaldehyde detoxifying enzymes mentioned above as well as of enzymes involved in further downstream catabolism was found in this study, whereby in the human system most formate enters the one-carbon cycle via cytoplasmic and mitochondrial C-1-tetrahydrofolate synthases (MTHFD1 and MTHFD1L). Larger amounts of formate could lead to adverse effects due to accumulation as this metabolic route is slow in humans compared to rats. Slow formic acid oxidation can cause accumulation of the acid and finally lead to anaerobic glycolysis and lactic acidosis^38^. Of note, no significant changes of expression levels of lactate dehydrogenases could be observed in our study. In addition, the exposure concentrations used in this study are very low. For the 0.1 ppm exposure experiment, the accumulated formaldehyde concentrations in the cell-free medium wells were in the range of endogenous formaldehyde concentrations detected in human blood^40^,^41^. To summarize, at least from gene expression level there was no indication for an adaptive response to potentially potentially accumulated formic acid for both the 0.1 ppm and 0.5 ppm exposure, which points towards the importance of the protective function of mucus and surfactant.

To date, no biomarker has been identified that could be used as a specific target for formaldehyde and in particular for low-level exposures, most likely owing to the nonspecific attack of airborne formaldehyde on a number of cellular targets. We hypothesized that after 3 days of exposure we could observe regulation of genes that support adaptation and/or defense against formaldehyde-mediated insults, while other studies using comparable concentrations but shorter exposure times were not able to detect cellular responses. Low environmental concentrations of approximately 0.04 ppm formaldehyde applied for 30 minutes did not increase the production of inflammation markers chemokine (C-C motif) ligand 2 (CCL2) and interleukin 8 (IL8) in A549 and BEAS-2B without any additional sensitization^42^ and also in another study, exposure to 0.2 ppm formaldehyde for 1 hour had no effect on IL-8 in A549 cells^20^. In a repeated exposure experiment of up to three exposures of epithelial cells of nasal origin for 1 hour to concentrations of approximately 0.17 ppm formaldehyde in 24 h intervals, no impact of treatment on cell viability and unchanged levels of IL-6 was reported by Bardet *et al*., and only after the third treatment IL-8 levels were reduced compared to air exposed control cells^43^. These data indicate that only minor expression changes can be expected at these low treatment concentrations, and the number of false positives might be high because of noise in the biological and experimental system. To reduce the number of random or false positives by means of biological fluctuations owing to chamber and time effects both the contemporaneous cultivated cells in the reference chamber and the consecutively cultivated cells exposed to a 0 ppm atmosphere in the exposure chamber were included as controls for identification of those transcripts that are differentially expressed owing to formaldehyde exposure. Interestingly, only few transcripts are commonly regulated with both exposure concentrations (Fig. 5), whereas distinct expression profiles emerge for 0.1 ppm and 0.5 ppm formaldehyde treatments.

The functions affected by the common genes are heterogeneous, and do not point to one central regulated pathway. Fibroblast growth factor 7 (FGF7) signalling is involved in cell proliferation, differentiation and developmental processes. In addition, chemoattractant activity has been described, linking its regulation to immunological signalling cascades. In mice, fibroblast growth factor-7 (FGF7) was shown to be able to cause pulmonary inflammation when present over longer periods, but it can be cytoprotective during injury^44^. Of note, in the human system FGF7 was reported to be a susceptibility locus for chronic obstructive pulmonary disease, and increased lung tissue FGF7 expression was associated with worse measures of lung function^45^. In this study, FGF7 expression was downregulated by formaldehyde treatment with both concentrations. Other common transcript interfere with immunoregulatory cascades, too, such as transient receptor potential cation channel subfamily C member 4 associated protein (TRPC4AP), which affects nuclear factor kappa B signaling, and RAR-related orphan receptor C (RORC) which is involved in innate lymphoid cell differentiation. Dysregulated expression of the transcription factors RORC and forkhead box p3 (FOXP3) in CD4 T cells of young asthmatic children was suggested to be associated with the sustained inflammatory process, whereby expression of RORC was up- and FOXP3 downregulated compared to healthy controls^46^. Formaldehyde exposure lowered RORC expression with both treatment concentrations.

Killer cell lectin-like receptor subfamily B, member 1 (KLRB1) has been investigated largely in immune cells as this receptor is expressed on most natural killer (NK) and subsets of T cells. KLRB1 likely acts as stimulatory molecule and is involved in the regulation of cross-talk between NK and antigen presenting cells, but the detailed signaling pathway is still insufficiently understood, in particular regarding the human system^47^,^48^. Cadherin EGF seven-pass-G-type-receptor 1 (CELSR1) is a non-classic cadherin that resides in the plasma membrane and is potentially involved in contact-mediated communication^49^,^50^. In the mouse, it is implicated in branching morphogenesis during normal lung development^51^. CELSR1 expression was negatively affected upon formaldehyde exposure in the A549 cells with both treatment concentrations. ST6 N-acetylgalactosaminide alpha-2,6-sialyltransferase 4 (ST6GALNAC4) exists either in a soluble form or is found in the Golgi where it is involved in sialic acid metabolism. In a mouse model of lung adenocarcinoma metastasis, upregulation of St6GalNAc4 glycosyltransferase in cancer cells contributed to aberrant glycosilation pattern which is suggested to be impicated in the promotion of metastasis^52^. Likewise the other above mentioned genes, except KLRB1, ST6GALNAC4 expression was reduced by formaldehyde.

Limited functional information is available for the other common transcripts. Kinesin family member 6 (KIF6), which was upregulated for both treatment concentrations in this study, is a microtubule associated motor protein involved in organelle transport and is predicted to have a function in cilia assembly^53^. Chromosome 11 open reading frame 54 (C11 or f54) encodes a protein with ester hydrolase activity, which is localized in the nucleus and in the exosomes, but further information on its function is limited^54^. The noncoding gene DIO3OS (DIO3 Opposite Strand/Antisense RNA (Head To Head)) regulates the expression of iodothyronine deiodinase type III (DIO3), which catalyzes the inactivation of thyroid hormone^55^. Zinc finger protein 507 (ZNF507) is most likely involved in transcriptional regulation. LOC101929153 and LOC 101928132 are RNA genes affiliated with the ncRNA class and the function of FLJ31945 is unknown.

To assess the biological functions affected by formaldehyde exposure in more detail, the 0.1 ppm and 0.5 ppm exposure profile were further analyzed separately. We used a generic GO Term mapper and enrichment of different GO biological processes to assess involved biological functions. The distinct gene sets mapped to similar functional processes in a generic GO Slim term analysis, such as cellular nitrogen compound metabolism, signal transduction, and biosynthetic processes; functions associated with the modification of cellular proteins, anatomical structure developments, transport and differentiation; and processes affecting the immune system, cell death and stress response. However, functional differences became more obvious when closely assessing the functions for significant enrichment of different GO biological processes. Specific metabolic, lipid biosynthetic and lung development-associated functions are affected by lower concentration, whereas proliferative and apoptotic processes dominate with the higher exposure (Fig. 4D).

It should be mentioned that a potential limitation for the investigations of biological functions and processes with low-dose treatments is that molecular interactions recorded in databases were typically characterized in diseased stages or situations of stress, and only a few pathways are also described in a low level context where non-monotonic and hormetic responses become important^56^,^57^.

Results of this study point towards a potential role of lipid biochemical pathways as part of the cell’s response to formaldehyde. The surfactant film that lines the alveolar surface, which is composed to 90% of lipids, is a critical component of the lung innate immune system, in addition its protective function from high surface tension^58^. Similarly, AE type II A549 cells synthetize lecithins with a high percentage of disaturated fatty acids, resulting in a phospholipid pattern typical for pulmonary surfactant-producing cells^23^. 0.1 ppm formaldehyde exposure interfered with several processes involved in lipid metabolism, including the synthesis of phosphatidic acid, an intermediate in phospholipid biosynthesis and important precursor for surfactant synthesis^59^, and the biosynthesis of neutral lipids, triglyceride and acylglycerol. Furthermore, processes involved in the biosynthesis of phosphatidylcholine, the main surfactant phospholipid, are affected by both exposure concentrations. In addition, KLRB1, which is also regulated by both concentrations, interacts with acid sphingomyelinase, an enzyme that catalyzes the hydrolysis of sphingomyelin. Sphingomyelin is a minor phospholipid surfactant component, but some of its precursors and catabolites have important biological roles in lung structure and function^59^, e.g., ceramide is a lipid second messenger involved in programmed cell death, differentiation and proliferation^47^. Such processes were associated with 0.5 ppm formaldehyde exposure in our study, but further investigations are needed to demonstrate any functional link to surfactant remodelling.

In conclusion, *in vitro* exposure models are a valuable support for the toxicological assessments of volatile compounds. In addition to acute reactions, now also longer, adaptive responses can be investigated, which so far could be approximated only via repeated exposure experiments. When trying to generate low-concentration atmospheres with reactive water-soluble chemicals such as formaldehyde, special attention must be paid to maintain a highly humidified atmosphere to guarantee cell vitality, while avoiding condensation. Transcriptional analysis of cellular responses showed that potential exposure biomarkers and concentration specific response pattern can be identified at sublethal concentrations when using adequate controls. Although the results of this study cannot be extrapolated directly to *in vivo* settings, changes of surfactant composition could represent a potential sensitive target that should be further investigated given that dysregulation of these functions can affect lung maturation and development and lead to respiratory illnesses^58^,^59^, and both tobacco smoke, a number of aerosols and gases were shown to exert toxicity by interacting with pulmonary surfactant^60^,^61^.

## Methods

### Exposure incubator system

Main components of the exposure incubator were the dosing unit, the humidifying unit, the gas transfer lines and the incubators containing exposure and reference chambers. All components were integrated into a modular aluminium profile system (BFM, Hirtenberg, Austria). The dosing unit was composed of the dosing vessel (Bioenergy 2020+, Graz, Austria), the liquid mass flow controller (Bronkhorst, Ruurlo, The Netherlands) for injection of the liquid standard into a glass tube system (Ruprechter, Innsbruck, Austria) located inside an aluminium evaporator block with a heating cartridge (Bioenergy 2020+, Graz, Austria). The humidifying unit consisted of a water bubbling system (glass flasks, Ruprechter, Innsbruck, Austria) and peristaltic pumps (VWR, Vienna, Austria). The water bubbling system was tempered by circulating thermostats (Lauda, Lauda-Königshofen, Germany). The gas flows were purified with heated sterile filters (200 °C) (Kaeser Kompressoren, Coburg, Germany) and regulated with gas mass flow controllers (Vögtlin, Lyss, Switzerland). Gas transfer lines were heated with heating bands (Lohmann, Graz, Austria). Incucell incubators (VWR, Vienna, Austria) were used to temper the exposure and reference chambers. Exposure and reference chambers were composed of polypropylene; lids were composed of polyvinyl chloride, and also served as inspection windows. The boxes contained aluminum inserts and fans (Bioenergy 2020+, Graz, Austria) to enable the circulation of the gas stream. Chambers were equipped with temperature sensors (PMR, Graz, Austria) at two positions, and the exposure chamber was additionally equipped with a humidity sensor (E + E Elektronik, Engerwitzdorf, Austria). The equipment for the switching cabinet was purchased from Siemens-Sonepar (Graz, Austria). Data logging and software for the process control system were obtained from Gantner Instruments (Schruns, Austria).

Apart from maintaining exact culture conditions as mentioned in the main text, the exposure incubator system, including all gas transfer lines, can be sterilized by dry heat (130 °C) to guarantee sterile culture conditions.

### Cell culture

A549 cells (Cell Lines Service, Eppelheim, Germany) were derived from a human lung carcinoma. Standard submerged cultures were maintained in Ham’s F12/DMEM (Sigma-Aldrich, St. Louis, MO, USA) supplemented with 10% (v/v) fetal calf serum for regular cultures. For ALI cultures, 100 μl of a cell suspension containing 50,000 A549 cells/mL were seeded in each transwell insert (24-well format, 0.4 μm pore polyester membrane inserts, #3470, Corning Life Sciences, Kaiserslautern, Germany), and bottom wells were filled with 600 μL of standard culture medium. Medium was removed and cells were reefed after 3 days. Cells were grown to confluence until day 7. On day 10, the bottom medium was changed, and medium was removed gently from the dense cell layer in the upper well. Cultures were maintained for 3 days in the standard cell culture incubator to adapt to ALI conditions, and morphology was checked daily. To prepare ALI cultures for the experiment, fresh 24-well plates were pre-filled with medium and covered with a silicon mat with punched holes for the transwell inserts (Figs 1D and 4A). Because of the silicon interlayer, each well had to be filled with 2.4 mL to guarantee that the bottom of the transwell insert reached the medium. The silicon mat blocked any direct contact of the analyte gas with the medium. ALI cultures were placed in the center rows of the 24-well plates.

### Viability assay

Liquid formaldehyde treatments: 100 μL of a cell suspension containing 100,000 A549 cells/mL were seeded in each well of a 96-well plate, and grown for 3 days to confluence. Cells were then treated with formaldehyde (addition of a 100 mM stock solution in medium) at concentrations ranging from 0.100 to 6.250 mM, for 24, 48, and 72 h. To measure cell viability, 10% (v/v) of CellTiter-Blue reagent (Promega, Mannheim, Germany) was added to the cells, and fluorescence was determined at 544/590 nm using a Fluoroskan Ascent FL plate reader (Thermo Fisher Scientific, Waltham, MA, USA) after 2 h of incubation. CellTiter-Blue reagent is a resazurin dye, which is irreversibly reduced to a highly red fluorescent resorufin, mainly because of NAD(P)H dehydrogenase activity. The half maximal (50% inhibitory) concentration (IC_50_) was calculated using the original concept of Chou and Talalay^62^ with the CalcuSyn software (Biosoft, Cambridge, UK). Volatile formaldehyde exposures: ALI cultures of A549 cells were exposed to gaseous formaldehyde for 72 h. Inserts with ALI cultures were then placed in fresh medium containing 10% (v/v) of CellTiter-Blue reagent and incubated for 4 h before fluorescence measurements.

### Gene expression analysis

RNA isolation: RNA isolation was performed using the NucleoSpin RNA/Protein isolation kit (Macherey-Nagel, Düren, Germany) according to the manufacturer’s instructions. Affymetrix HG-U133 Plus 2.0 arrays:Microarray analysis was performed at ATLAS Biolabs GmbH, Berlin, Germany. In brief, labeling was achieved by using the Applause 3′-Amp System and Encore Biotin Module (NuGEN, Leek, The Netherlands). Hybridization was performed according to standard protocols for Affymetrix HG-U133 Plus 2.0 arrays. Fluidics washing was done using the Affymetrix Wash and Stain Kit (#900720), protocol FS450_0004. Arrays were scanned with Affymetrix Gene ChIP Scanner 3000 7 G, and images were processed using the Expression Console. Data are stored at the GEO repository (GSE76790). Bioinformatics: Raw data were analyzed with R/Bioconductor version 3.0.1 using Affymetrix R-package^63^ with default analysis settings, and normalized using MAS 5.0 (mas5) and log2-transformed. To test for differential expression between two conditions, eBayes function from limma package in R was used^64^. To identify differentially expressed candidate genes, the *p*-value threshold was set to 0.05 (Figures in supplementary information was based on threshold of *p*-value <0.5 and fold change 2; here the fold-change is the ratio of the mean normalized intensities for the two conditions). The probesets/transcript clusters were annotated using hgu133plus2.db package in R. To identify genes that were shared in the two comparisons, differentially expressed genes with *p*-value <0.05 in both comparisons were filtered. Venn diagrams were created based on the shared genes. GO biological processes enrichment for differentially expressed transcripts were performed using topGO package in R using classic count Fisher test^65^. A biological process was considered significant when classic score (p value) <0.01. GOTermMapper (http://go.princeton.edu/cgi-bin/GOTermMapper) function was used to map generic Gene Ontology Term (biological process, function and component).

An additional approach to find common genes for 0.1 and 0.5 ppm exposure was done using two-way analysis of variance (ANOVA)^66^ without interactions to model the factors treatment (levels: no treatment, 0.1 ppm and 0.5 ppm FA) and chamber (levels: reference and control chamber) in the experiment. The effect of the treatment was determined on the expression of each gene. The ANOVA resulted in 198 probesets with significant group differences in the expression among the three conditions (*p*-value <0.005). Post-hoc Tukey’s HSD test^67^ and template matching^68^ on the genes which have been selected based on ANOVA was applied. To identify candidate genes that are differentially expressed in both formaldehyde treatments compared to controls we filtered for genes that fulfilled the following criteria: (i) Tukey’s HSD test for 0.1 ppm treatment versus control with p-value <0.05, (ii) Tukey’s HSD test for 0.5 ppm treatment versus control with p-value <0.05, and (iii) the expression profile matches a template with a similar expression in the 0.1 and 0.5 ppm treatment samples and opposite expression in the controls with a FDR < 0.05 (Benjamini-Hochberg correction). This filtering procedure resulted in 29 probesets, 19 of them have a known gene annotation (Supplemental file S2, S2-Fig. 2).

### Formaldehyde analytics

#### Analysis of liquid samples

2,4-Dinitrophenylhydrazine (DNPH; Sigma-Aldrich, Vienna, Austria) was used for the derivatization of the formaldehyde in medium samples^69^. Briefly, cell culture media was incubated 1:4 with a 5 mM DNPH solution prepared in acetonitrile (Sigma-Aldrich, Vienna, Austria) containing 10 mM perchloric acid (final concentration). Precipitated protein was removed by centrifugation prior injection to HPLC. Formaldehyde-2,4-dinitrophenylhydrazone TraceCERT^®^ (Sigma-Aldrich, Vienna, Austria) was used as calibration standard.

Separations were performed on an Agilent Technologies 1100 HPLC system (Waldbronn, Germany) using a LiChrospher^®^ 100 RP-18 (5 μm) LiChroCART^®^ 250-4 column (l = 250 mm, d = 4 mm; Merck, Darmstadt, Germany). The mobile phase comprised acetonitrile HiPerSolv CHROMANORM HPLC grade; VWR, Radnor, PA) and water 1:1 (v/v), elution was performed in isocratic mode for 10 minutes. The DAD was set to 360 nm and the flow rate, sample volume and column temperature were adjusted to 2 ml/min, 10 μl and 25 °C, respectively. Analysis of airborne formaldehyde: The formaldehyde measurements were made using an Aerolaser 4021 (AL4021, AERO-LASER GmbH, Garmisch-Partenkirchen, Germany), an automated and continuously working formaldehyde (HCHO) analyzer for gaseous and liquid samples with a stated detection rage of 100 ppt–50 ppm (gas)^70^. For the analysis, the formaldehyde is absorbed in an aqueous solution, which then reacts with acetylacetone in the presence of ammonia (Hantzsch reaction). The final product, 3,5-diacetyl-1,4-dihydrolutidine (DDL) can be detected by its fluorescence (410(ex)/510 (em)nm). The AL4021 was calibrated in the range from zero level to 6.2 ppm formaldehyde using a freshly purchased certified liquid (aqueous) standard (formaldehyde IC standard, Sigma-Aldrich, Vienna, Austria).

## Additional Information

**How to cite this article**: Gostner, J. M. *et al*. Cellular reactions to long-term volatile organic compound (VOC) exposures. *Sci. Rep.* **6**, 37842; doi: 10.1038/srep37842 (2016).

**Publisher's note:** Springer Nature remains neutral with regard to jurisdictional claims in published maps and institutional affiliations.

## Supplementary Material

Supplementary Information

Supplementary Dataset S3

## Figures and Tables

**Table 1:** Dissolved formaldehyde and remaining medium after 3 days of exposure (n = 3 for each treatment).

condition		formaldehyde intake [μM]		Remaining medium [%]			
		exposure chamber		exposure chamber		reference chamber	
		mean	s.d.	mean	s.d.	mean	s.d.
0.1 ppm	medium	36.40	2.07	93.54	1.81	89.81	3.67
	ALI cultures	1.68	0.50	95.57	2.00	93.62	1.49
0.5 ppm	medium	206.44	12.87	88.64	4.90	86.15	4.93
	ALI cultures	4.06	1.86	92.59	4.45	90.05	3.15
0 ppm	medium	—	—	93.10	1.90	91.70	2.38
	ALI cultures	—	—	96.30	1.05	95.52	2.30

Formaldehyde intake was measured in wells containing medium only and in wells with A549 ALI cultures; cell layers prevented the transfer of airborne formaldehyde to the underlying medium.
