# Supplementary material for: Cellular reactions to long-term volatile organic compound (VOC) exposures
Source: Sci Rep. 2016 Dec 1;6:37842. doi: 10.1038/srep37842 (PMC5131358; doi:10.1038/srep37842)

Supplemental file S1

Manuscript title

Cellular reactions to long-term volatile organic compound (VOC) exposures

Authors

Johanna M Gostner[1*], Johannes Zeisler[1,2,†], Mohammad Tauqeer Alam[3], Peter Gruber[1], Dietmar Fuchs[4], Kathrin Becker[4], Kerstin Neubert[5,††], Markus Kleinhappl[2], Stefan Martini[2], Florian Überall[1]

Affiliations

[1] Division of Medical Biochemistry, Biocenter, Medical University of Innsbruck, Austria

[2] Bioenergy 2020+, Graz, Austria

[3] Division of Biomedical Sciences, Warwick Medical School, University of Warwick, UK

[4] Division of Biological Chemistry, Biocenter, Medical University of Innsbruck, Austria

[5] ATLAS Biolabs GmbH, Berlin, Germany

† current address: Energy and Environmental Technology Group, Fritz Egger GmbH & Co. OG, St. Johann in Tirol, Austria

†† current address: Department of Mathematics and Computer Science, Freie Universität Berlin, Germany

Supplemental file 1 - process control, evaporation and formaldehyde concentration

Process control: Process control data were continuously monitored in one minute intervals during the exposure experiments. Experiments are listed in Table 1 (main text). Relative humidity was measured in the exposure chamber only. Temperatures and gas flow was monitored in both chambers.

Evaporation and formaldehyde uptake: 24-well plates were equipped as following: The middle rows were loaded with 2.6 mL medium and transwell inserts containing A549 ALI cultures, the other rows were filled with 2.6 mL medium only. After 3 days of exposure, the amount of evaporated media was estimated by weighting the remaining medium in the wells. Formaldehyde uptake was measured in the wells that contained medium only, and in those that contained A549 cultures to determine whether formaldehyde passes through the cell layer and accumulates in the medium below. For a number of experiments, the formaldehyde concentration in the headspace of the exposure chamber was measured.

S1-Fig. 1: Exposure to 0.1 ppm formaldehyde, experiment no. 1.

Process control data were recorded continuously during the experiment and include (A) temperatures in the exposure chamber (EC, blue line) and in the reference chamber (RC, green line), (B) relative humidity in the exposure chamber and (C) gas flows (HCS: humidified carrier stream; VCS: vaporized carrier stream). Evaporated medium is shown as percentage of initial filing level of the individual wells of a 24-well plate of the exposure (upper D) and of the reference chamber (lower D) after 72 h of exposure. At the end of the experiment, the amount of dissolved formaldehyde [µM] in medium was measured in selected wells (E). In addition, the concentration in the atmosphere of the exposure chamber was measured (F).

S1-Table 1: Evaporated media (% of initial volume) in the exposure (A) and the reference chamber (B) in each individual well of a 24-well plate after 72 h of exposure (grey = wells with transwell inserts, white = medium filing only). The amount of dissolved formaldehyde [µM] in the medium is shown in (C).

(A) Exposure chamber

Amount of evaporated medium
[% of initial volume]

D	C	B	A	
8.03	7.91	7.72	8.36	1
6.70	6.19	5.62	6.50	2
3.43	3.04	2.55	1.71	3
3.52	2.90	2.02	2.42	4
5.51	3.86	3.82	5.61	5
7.15	5.08	4.52	6.38	6

(B) Reference chamber

Amount of evaporated medium
[% of initial volume]

D	C	B	A	
10.57	10.81	9.38	9.22	1
10.70	9.38	9.40	8.96	2
5.14	5.04	4.88	6.23	3
5.68	5.12	4.16	6.11	4
6.77	5.67	4.93	8.63	5
10.05	8.90	8.69	8.72	6

(E) Exposure chamber

Formaldehyde [µM]

D	C	B	A	
				1
38.01	37.84	34.81	37.16	2
1.35	0.84	1.01	1.18	3
				4
1.35	1.35	1.35	37.67	5
36.16	36.16	37.67	39.52	6

S1-Fig.1: 0.1 ppm exposure, no.1

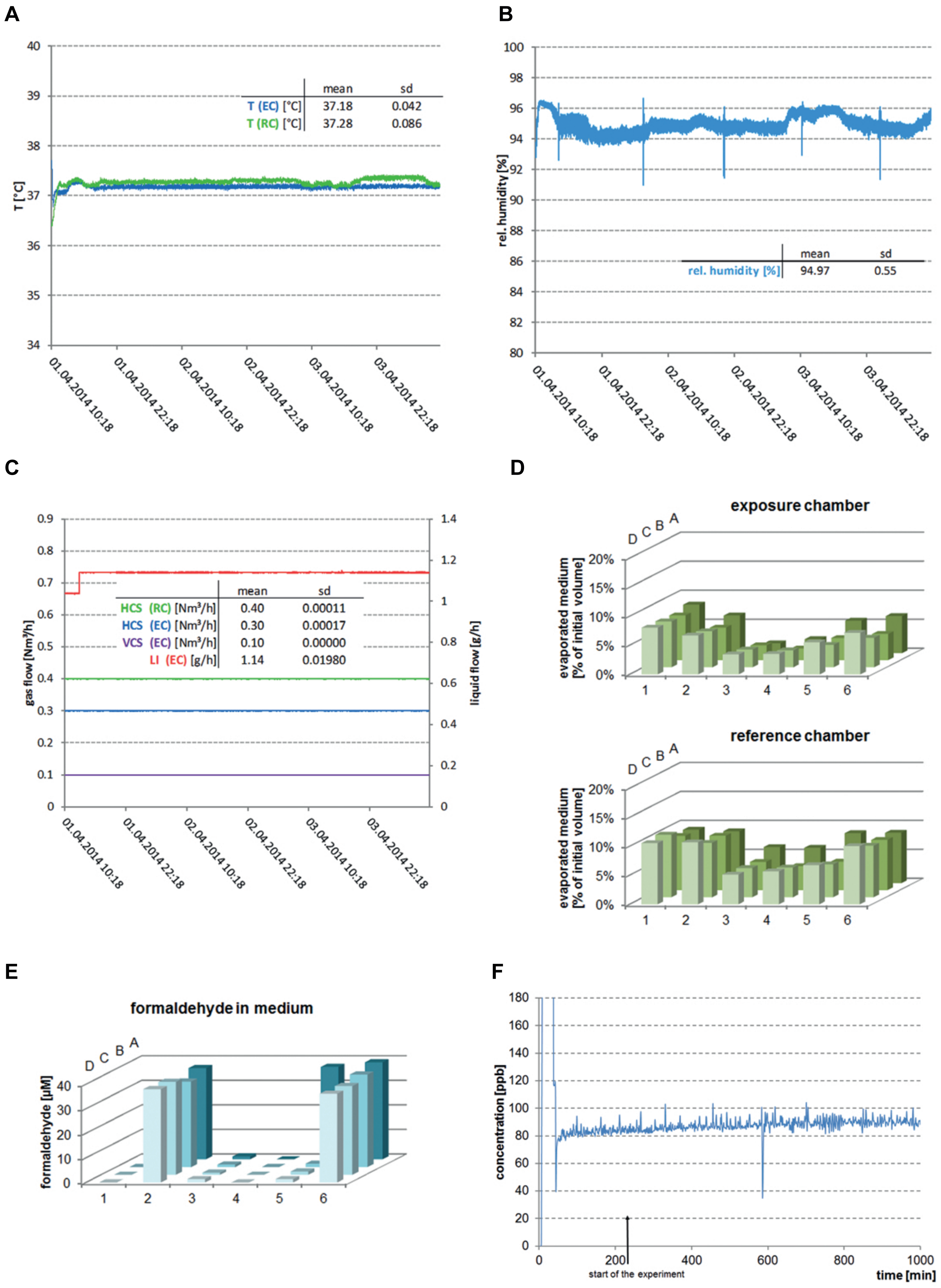

S1-Fig. 2: Exposure to 0.1 ppm formaldehyde, experiment no. 2.

Process control data were recorded continuously during the experiment and include (A) temperatures in the exposure chamber (EC, blue line) and in the reference chamber (RC, green line), (B) relative humidity in the exposure chamber and (C) gas flows (HCS: humidified carrier stream; VCS: vaporized carrier stream). Evaporated medium is shown as percentage of initial filing level of the individual wells of a 24-well plate of the exposure (upper D) and of the reference chamber (lower D) after 72 h of exposure. At the end of the experiment, the amount of dissolved formaldehyde [µM] in medium was measured in selected wells (E). In addition, the concentration in the atmosphere of the exposure chamber was measured (F).

S1-Table 2: Evaporated media (% of initial volume) in the exposure (A) and the reference chamber (B) in each individual well of a 24-well plate after 72 h of exposure (grey = wells with transwell inserts, white = medium filing only). The amount of dissolved formaldehyde [µM] in the medium is shown in (C).

(A) Exposure chamber

Amount of evaporated medium
[% of initial volume]

D	C	B	A	
8.51	7.01	6.58	8.57	1
5.88	3.40	6.07	6.45	2
6.30	3.86	4.46	3.49	3
4.20	2.47	4.81	4.93	4
5.13	4.10	3.40	4.13	5
4.51	3.90	4.76	5.55	6

(B) Reference chamber

Amount of evaporated medium
[% of initial volume]

D	C	B	A	
13.53	11.92	11.14	11.74	1
12.04	10.65	11.26	10.04	2
8.27	5.77	5.69	11.15	3
8.65	6.41	6.68	6.68	4
7.62	6.42	7.38	6.88	5
10.12	13.62	8.65	10.83	6

(D) Exposure chamber

Formaldehyde [µM]

D	C	B	A	
				1
39.18	30.61	35.32	36.83	2
2.01	2.53	2.19	2.02	3
				4
2.36	2.35	2.19	2.19	5
33.47	34.48	39.01	39.35	6

S1-Fig.2: 0.1 ppm exposure, no.2

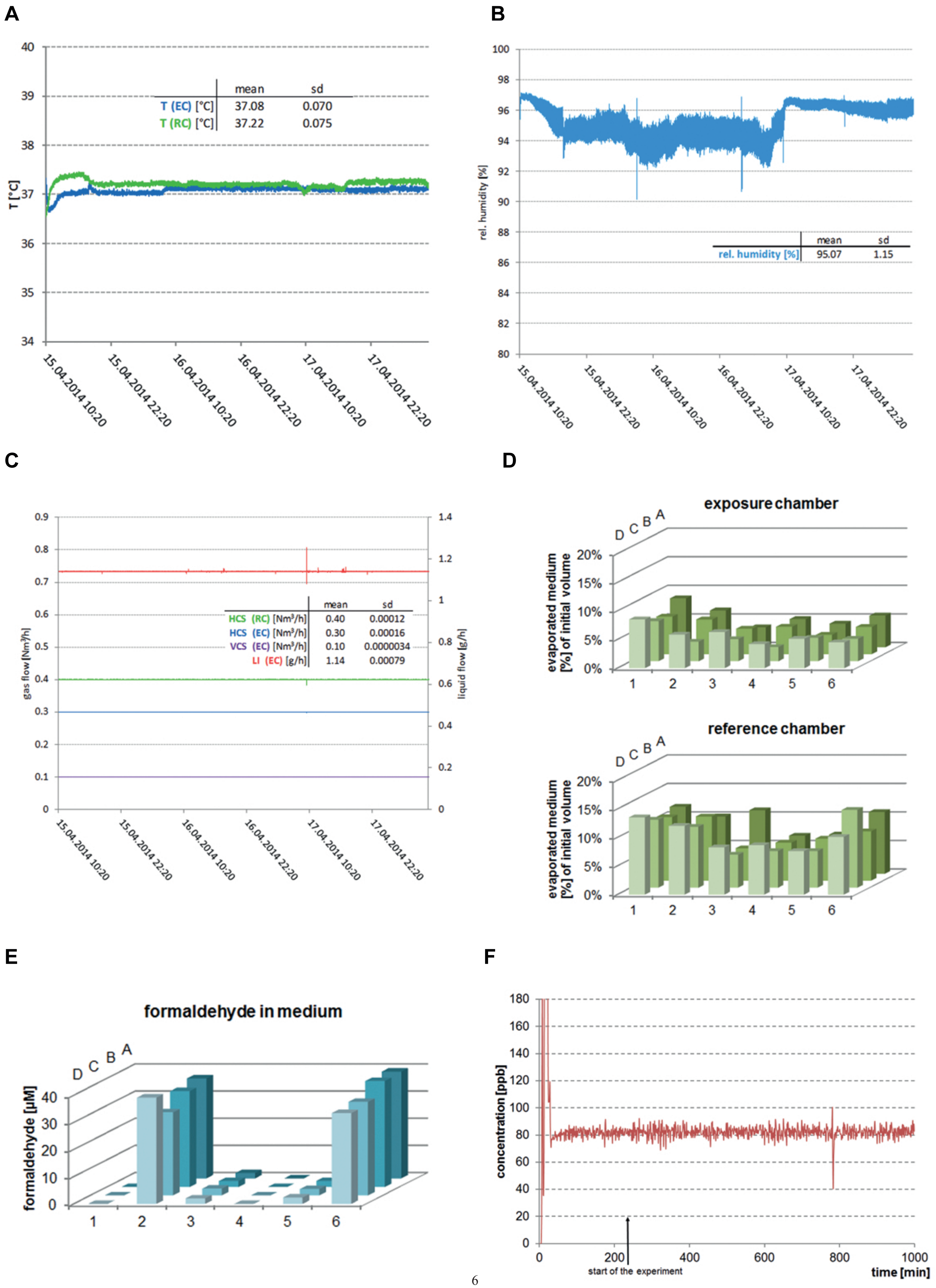

S1-Fig. 3: Exposure to 0.1 ppm formaldehyde, experiment no. 3.

Process control data were recorded continuously during the experiment and include (A) temperatures in the exposure chamber (EC, blue line) and in the reference chamber (RC, green line), (B) relative humidity in the exposure chamber and (C) gas flows (HCS: humidified carrier stream; VCS: vaporized carrier stream). Evaporated medium is shown as percentage of initial filing level of the individual wells of a 24-well plate of the exposure (upper D) and of the reference chamber (lower D) after 72 h of exposure. At the end of the experiment, the amount of dissolved formaldehyde [µM] in medium was measured in selected wells (E). In addition, the concentration in the atmosphere of the exposure chamber was measured (F).

S1-Table 3: Evaporated media (% of initial volume) in the exposure (A) and the reference chamber (B) in each individual well of a 24-well plate after 72 h of exposure (grey = wells with transwell inserts, white = medium filing only). The amount of dissolved formaldehyde [µM] in the medium is shown in (C).

(A) Exposure chamber

Amount of evaporated medium [% of initial volume]

D	C	B	A	
9.00	8.15	8.31	8.88	1
0.00	6.92	6.69	7.96	2
6.08	4.65	4.73	6.35	3
13.04	4.58	4.50	6.04	4
5.65	5.00	5.92	6.50	5
6.88	6.77	8.31	6.77	6

(B) Reference chamber

Amount of evaporated medium [% of initial volume]

D	C	B	A	
11.15	9.19	10.54	10.27	1
10.27	8.38	30.15	8.31	2
8.15	6.96	5.15	4.15	3
6.96	5.73	5.77	5.31	4
8.77	6.85	6.69	7.15	5
9.04	6.92	7.15	6.58	6

(C) Exposure chamber

Formaldehyde [µM]

D	C	B	A	
				1
35.65	35.65	36.49	37.00	2
1.18	1.85	1.18	1.68	3
2.35	1.68	1.35	1.35	4
1.51	35.15	34.47	35.99	5
				6

S1-Fig. 3: 0.1 ppm exposure, experiment no.3

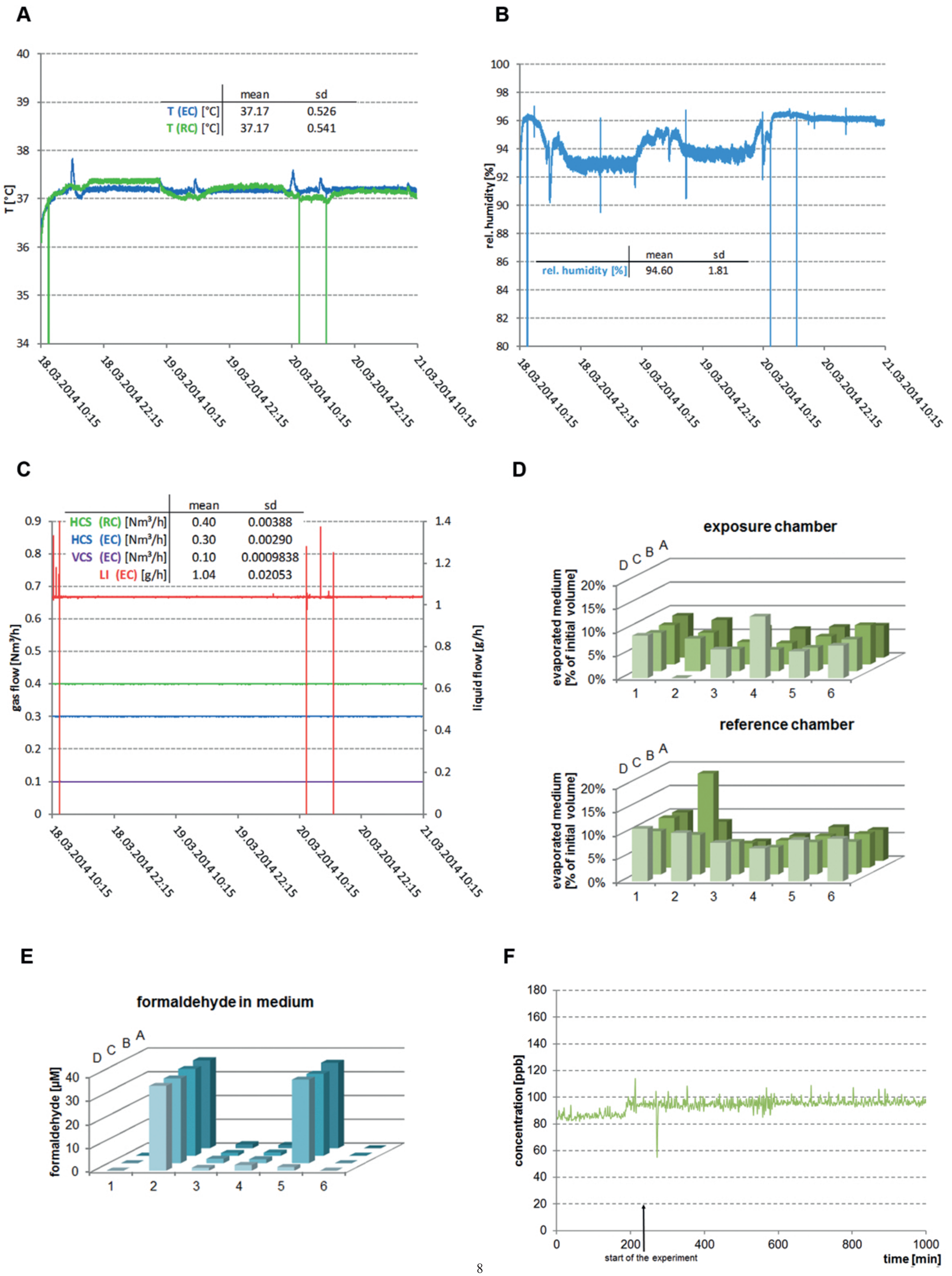

S1-Fig. 4: Exposure to 0.5 ppm formaldehyde, experiment no. 1.

Process control data were recorded continuously during the experiment and include (A) temperatures in the exposure chamber (EC, blue line) and in the reference chamber (RC, green line), (B) relative humidity in the exposure chamber and (C) gas flows (HCS: humidified carrier stream; VCS: vaporized carrier stream). Evaporated medium is shown as percentage of initial filing level of the individual wells of a 24-well plate of the exposure (upper D) and of the reference chamber (lower D) after 72 h of exposure. At the end of the experiment, the amount of dissolved formaldehyde [µM] in medium was measured in selected wells (E).

S1-Table 4: Evaporated media (% of initial volume) in the exposure (A) and the reference chamber (B) in each individual well of a 24-well plate after 72 h of exposure (grey = wells with transwell inserts, white = medium filing only). The amount of dissolved formaldehyde [µM] in the medium is shown in (C).

(A) Exposure chamber

Amount of evaporated medium [% of initial volume]

D	C	B	A	
6.80	6.35	6.87	7.27	1
7.73	6.18	6.79	7.04	2
6.50	5.44	6.36	7.63	3
6.32	6.38	5.82	6.47	4
7.57	7.34	7.59	8.43	5
11.43	10.88	10.48	12.07	6

(B) Reference chamber

Amount of evaporated medium [% of initial volume]

D	C	B	A	
8.54	7.97	8.16	8.35	1
8.04	7.42	7.85	8.58	2
5.09	4.31	4.20	5.07	3
5.75	5.09	5.88	4.88	4
9.74	9.40	9.47	10.36	5
13.43	14.01	16.35	16.12	6

(C) Exposure chamber

Formaldehyde [µM]

D	C	B	A	
				1
234.76	200.96	194.40	202.98	2
8.41	3.03	3.03	5.55	3
5.55	4.88	5.21	8.58	4
232.40	201.63	204.99	219.29	5
				6

S1-Fig.4: 0.5 ppm exposure, no.1

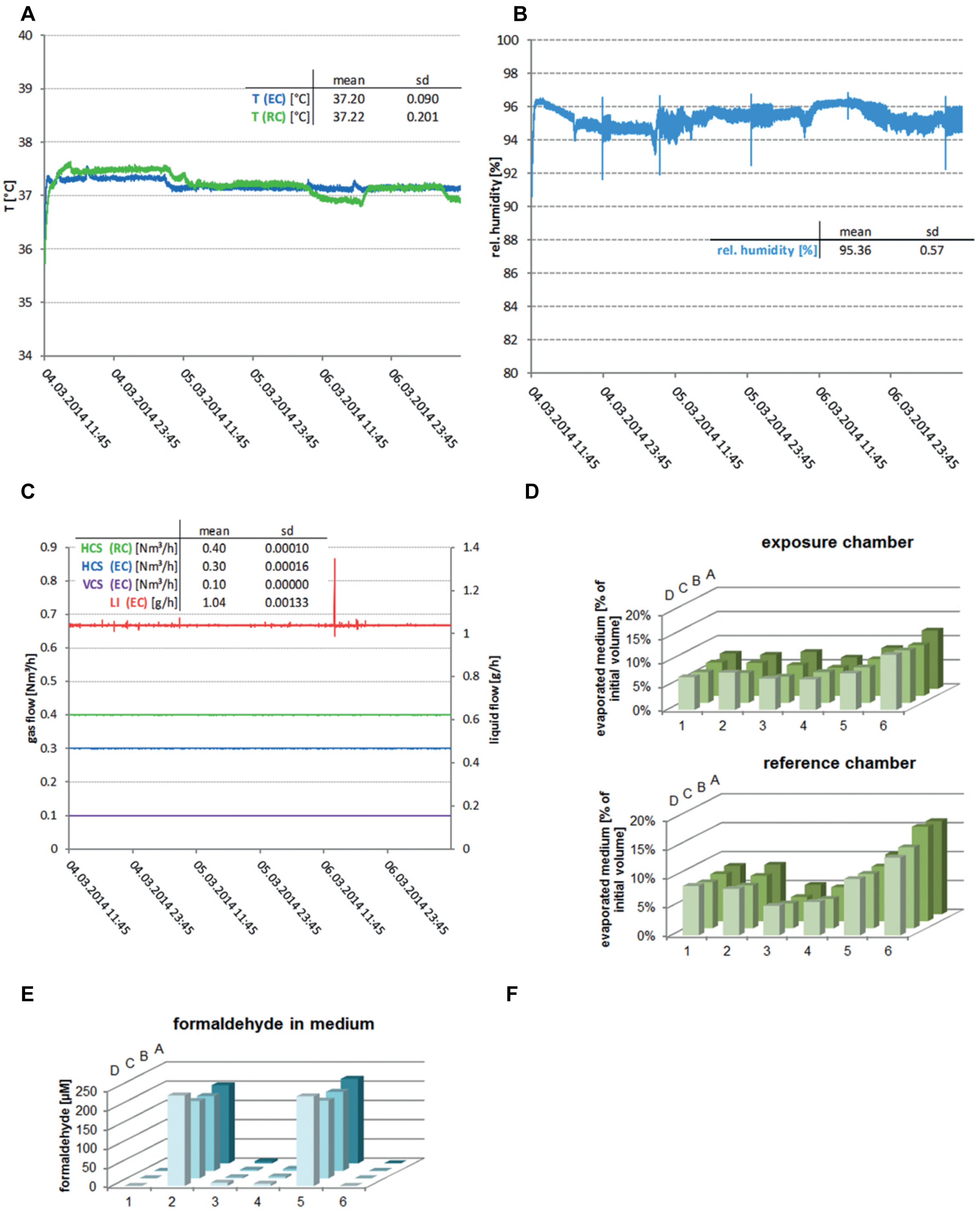

S1-Fig. 5: Exposure to 0.5 ppm formaldehyde, experiment no. 2.

Process control data were recorded continuously during the experiment and include (A) temperatures in the exposure chamber (EC, blue line) and in the reference chamber (RC, green line), (B) relative humidity in the exposure chamber and (C) gas flows (HCS: humidified carrier stream; VCS: vaporized carrier stream). Evaporated medium is shown as percentage of initial filing level of the individual wells of a 24-well plate of the exposure (upper D) and of the reference chamber (lower D) after 72 h of exposure. At the end of the experiment, the amount of dissolved formaldehyde [µM] in medium was measured in selected wells (E).

S1-Table 5: Evaporated media (% of initial volume) in the exposure (A) and the reference chamber (B) in each individual well of a 24-well plate after 72 h of exposure (grey = wells with transwell inserts, white = medium filing only). The amount of dissolved formaldehyde [µM] in the medium is shown in (C).

(A) Exposure chamber

Amount of evaporated medium [% of initial volume]

D	C	B	A	
8.06	7.93	7.91	9.23	1
7.46	6.68	6.84	14.46	2
6.58	5.40	4.91	4.91	3
5.84	5.15	4.52	5.36	4
7.81	4.34	4.99	6.72	5
7.65	7.02	7.23	14.86	6

(B) Reference chamber

Amount of evaporated medium [% of initial volume]

D	C	B	A	
12.92	11.04	10.32	10.62	1
10.98	10.47	10.27	9.17	2
8.41	7.52	7.54	7.23	3
7.31	8.23	7.50	7.08	4
11.33	6.48	5.24	6.15	5
11.49	9.08	10.04	10.00	6

(C)Exposure chamber

Formaldehyde [µM]

D	C	B	A	
				1
197.43	198.60	198.94	198.60	2
3.20	2.02	2.19	2.86	3
				4
				5
				6

S1-Fig.5: 0.5 ppm exposure, no.2

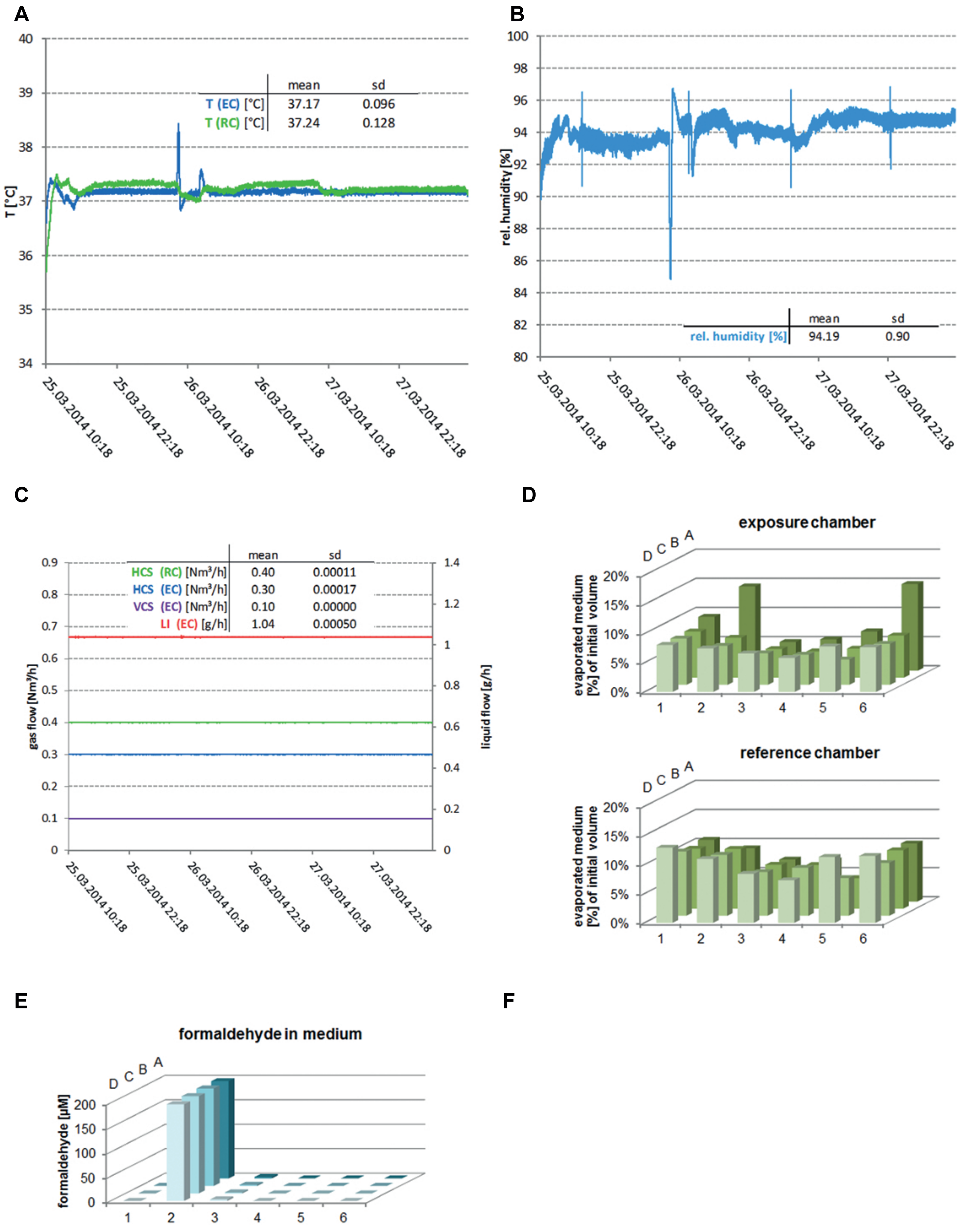

S1-Fig. 6: Exposure to 0.5 ppm formaldehyde, experiment no. 3.

Process control data were recorded continuously during the experiment and include (A) temperatures in the exposure chamber (EC, blue line) and in the reference chamber (RC, green line), (B) relative humidity in the exposure chamber and (C) gas flows (HCS: humidified carrier stream; VCS: vaporized carrier stream). Evaporated medium is shown as percentage of initial filing level of the individual wells of a 24-well plate of the exposure (upper D) and of the reference chamber (lower D) after 72 h of exposure. At the end of the experiment, the amount of dissolved formaldehyde [µM] in medium was measured in selected wells (E). In addition, the concentration in the atmosphere of the exposure chamber was measured (F).

S1-Table 6: Evaporated media (% of initial volume) in the exposure (A) and the reference chamber (B) in each individual well of a 24-well plate after 72 h of exposure (grey = wells with transwell inserts, white = medium filing only). The amount of dissolved formaldehyde [µM] in the medium is shown in (C).

(A) Exposure chamber

Amount of evaporated medium

[% of initial volume]

D	C	B	A	
7.17	6.21	6.68	6.37	1
6.00	4.00	6.27	5.71	2
2.81	4.48	2.96	2.69	3
4.08	3.59	4.10	5.16	4
3.69	3.65	3.11	3.55	5
5.37	5.97	5.36	4.98	6

(B) Reference chamber

Amount of evaporated medium

[% of initial volume]

D	C	B	A	
11.17	10.82	10.77	9.65	1
9.12	8.46	7.58	9.18	2
6.35	5.22	6.18	5.73	3
6.17	5.13	5.38	5.09	4
6.69	6.23	5.18	5.38	5
9.72	8.37	7.74	8.32	6

(C) Exposure chamber

Formaldehyde [µM]

D	C	B	A	
				1
210.04	180.44	211.22	214.24	2
4.04	2.86	3.03	3.87	3
				4
4.37	2.19	2.86	3.53	5
193.39	209.20	210.88	214.41	6

S1-Fig.6: 0.5 ppm exposure, no.3

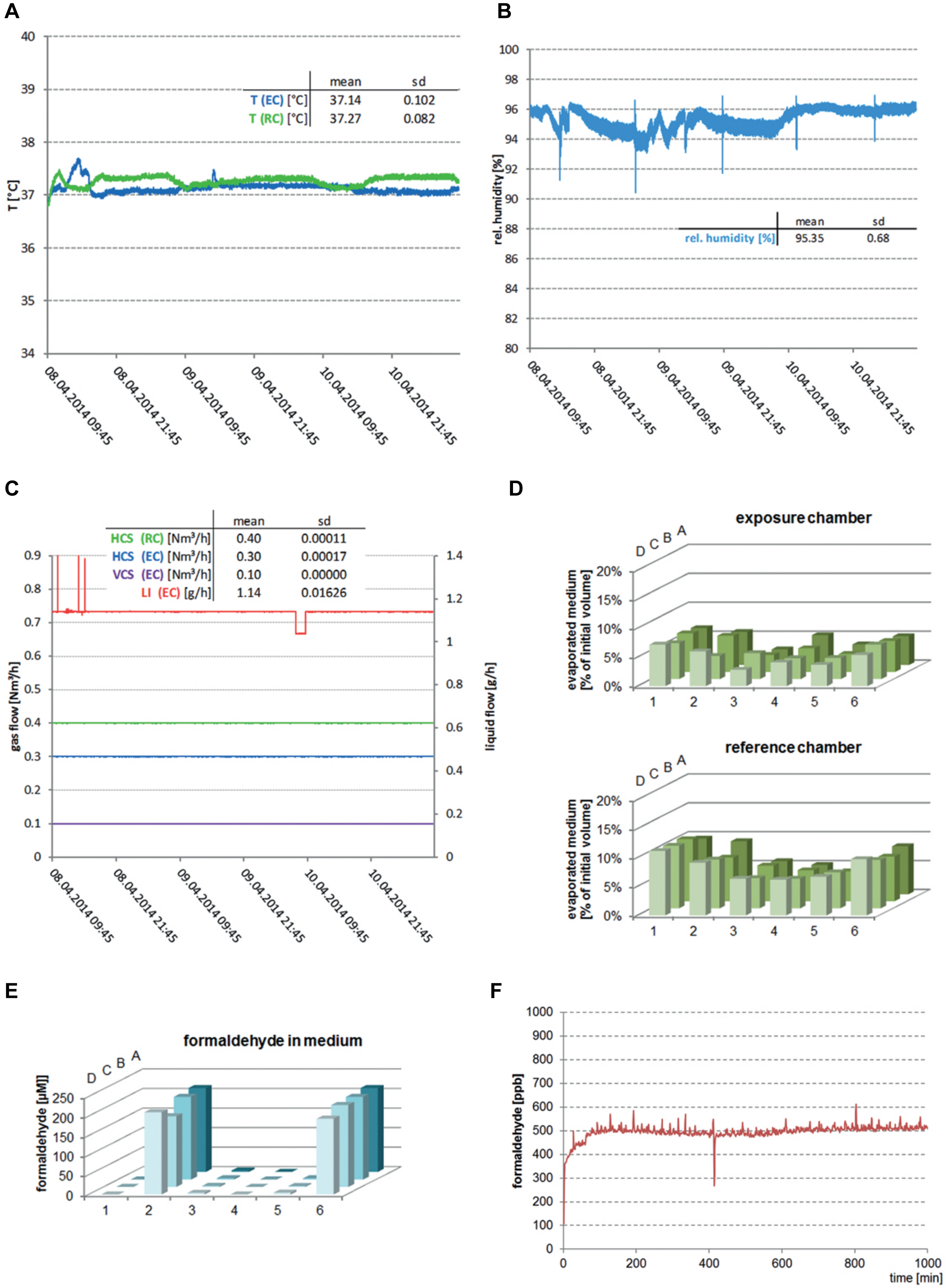

S1-Fig. 7: Exposure to 0.0 ppm formaldehyde, experiment no. 1.

Process control data were recorded continuously during the experiment and include (A) temperatures in the exposure chamber (EC, blue line) and in the reference chamber (RC, green line), (B) relative humidity in the exposure chamber and (C) gas flows (HCS: humidified carrier stream; VCS: vaporized carrier stream). Evaporated medium is shown as percentage of initial filing level of the individual wells of a 24-well plate of the exposure (upper D) and of the reference chamber (lower D) after 72 h of exposure.

S1-Table 7: Evaporated media (% of initial volume) in the exposure (A) and the reference chamber (B) in each individual well of a 24-well plate after 72 h of exposure (grey = wells with transwell inserts, white = medium filing only).

(A) Exposure chamber

Amount of evaporated medium
[% of initial volume]

D	C	B	A	
8.51	7.01	6.58	8.57	1
5.88	3.40	6.07	6.45	2
6.30	3.86	4.46	3.49	3
4.20	2.47	4.81	4.93	4
5.13	4.10	3.40	4.13	5
4.51	3.90	4.76	5.55	6

(B) Reference chamber

Amount of evaporated medium
[% of initial volume]

D	C	B	A	
13.53	11.92	11.14	11.74	1
12.04	10.65	11.26	10.04	2
8.27	5.77	5.69	11.15	3
8.65	6.41	6.68	6.68	4
7.62	6.42	7.38	6.88	5
10.12	13.62	8.65	10.83	6

S1-Fig.7: 0 ppm exposure, no.1

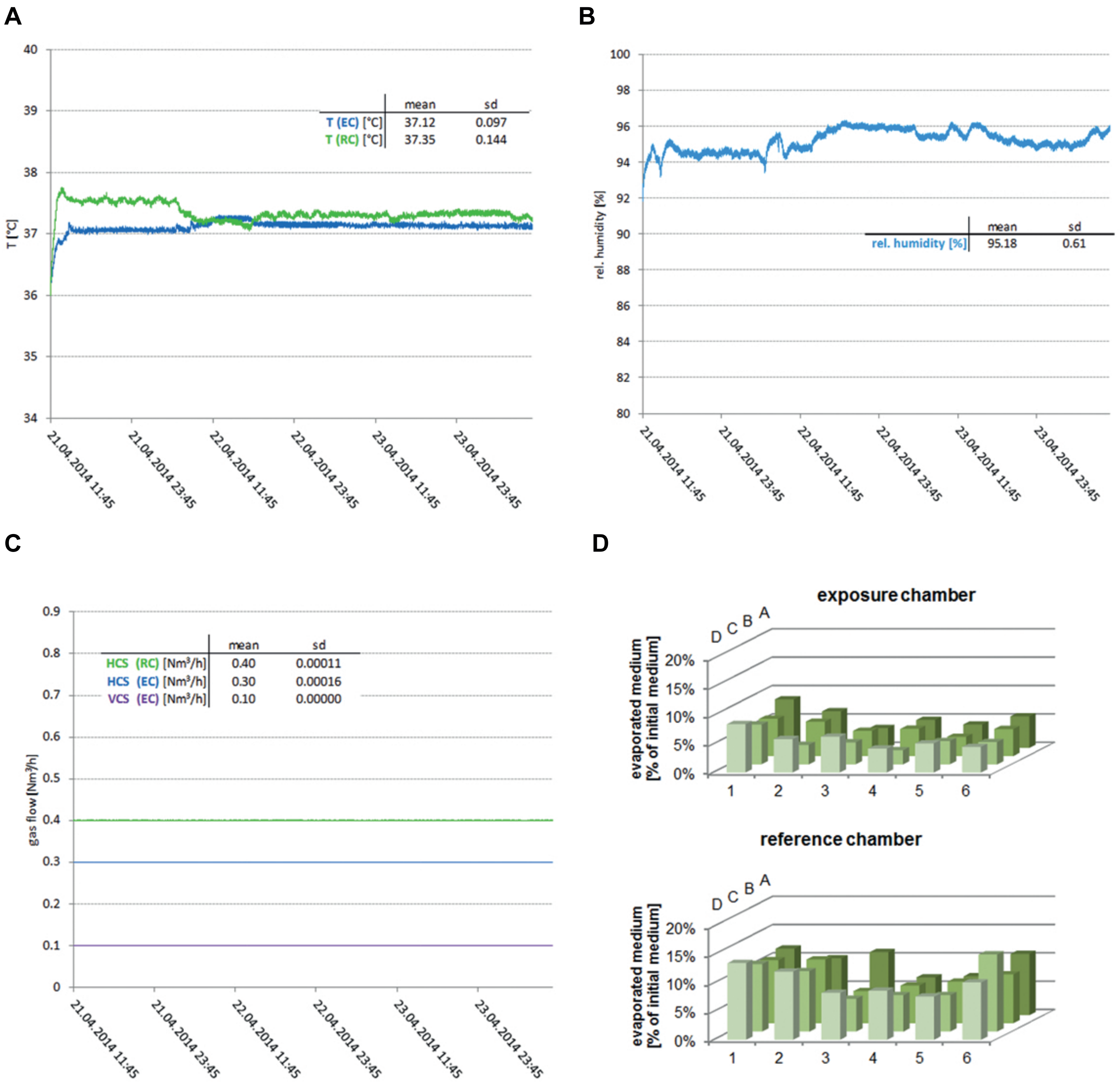

S1-Fig. 8: Exposure to 0.0 ppm formaldehyde, experiment no. 2.

Process control data were recorded continuously during the experiment and include (A) temperatures in the exposure chamber (EC, blue line) and in the reference chamber (RC, green line), (B) relative humidity in the exposure chamber and (C) gas flows (HCS: humidified carrier stream; VCS: vaporized carrier stream). Evaporated medium is shown as percentage of initial filing level of the individual wells of a 24-well plate of the exposure (upper D) and of the reference chamber (lower D) after 72 h of exposure.

S1-Table 8: Evaporated media (% of initial volume) in the exposure (A) and the reference chamber (B) in each individual well of a 24-well plate after 72 h of exposure (grey = wells with transwell inserts, white = medium filing only).

(A) Exposure chamber

Amount of evaporated medium
[% of initial volume]

D	C	B	A	
10.15	11.31	10.23	12.05	1
6.73	5.88	6.54	6.54	2
1.73	2.77	2.65	3.08	3
3.50	2.42	2.65	3.13	4
6.31	4.19	4.00	5.23	5
8.08	7.31	7.23	7.55	6

(B) Reference chamber

Amount of evaporated medium
[% of initial volume]

D	C	B	A	
9.05	9.73	8.46	11.15	1
9.00	6.68	6.35	6.49	2
3.12	2.42	3.15	3.19	3
3.85	3.26	3.01	3.28	4
8.64	5.23	5.03	4.88	5
11.29	6.47	7.03	7.13	6

S1-Fig.8: 0 ppm exposure, no.2

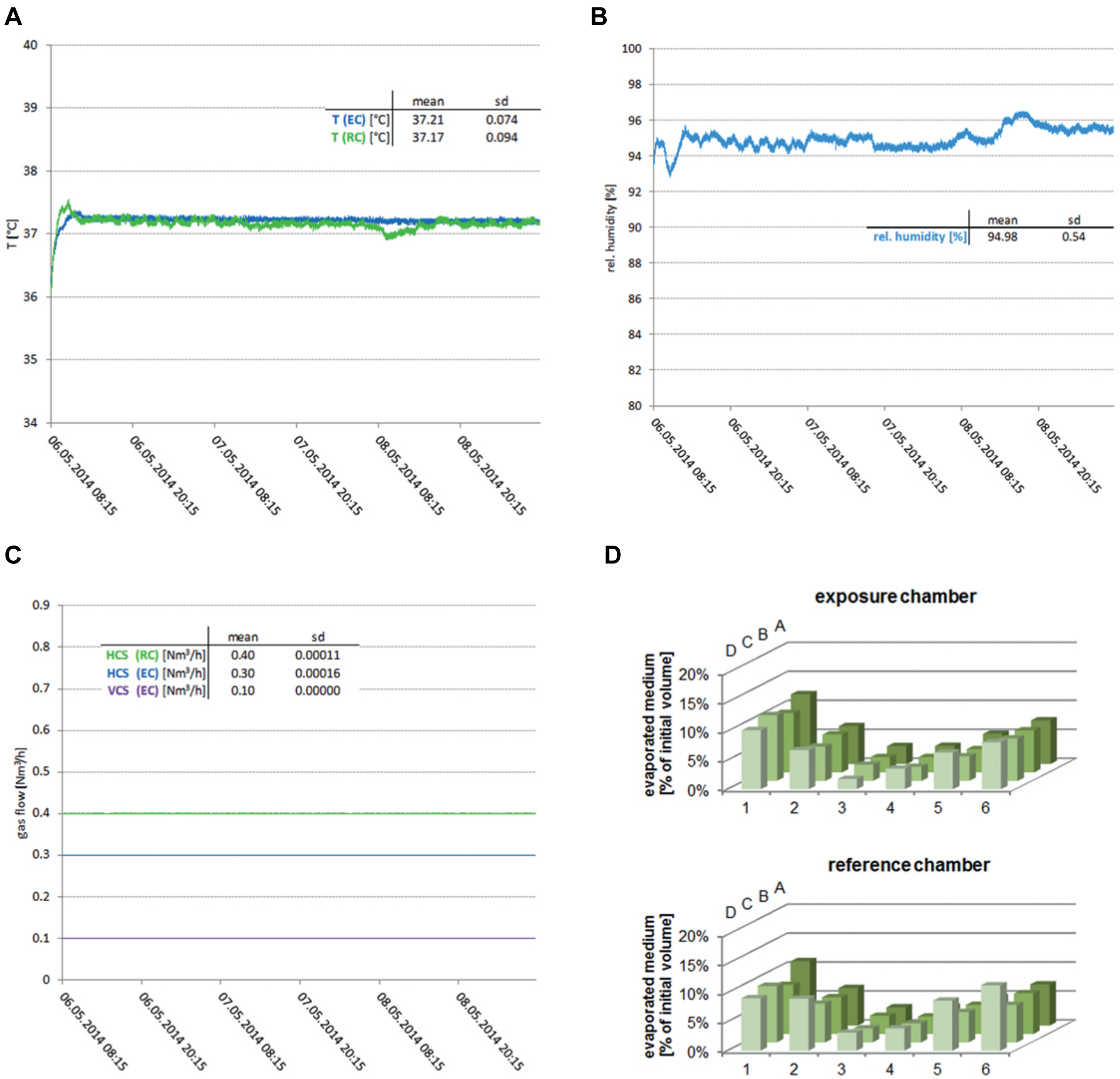

S1-Fig. 9: Exposure to 0.0 ppm formaldehyde, experiment no. 3.

Process control data were recorded continuously during the experiment and include (A) temperatures in the exposure chamber (EC, blue line) and in the reference chamber (RC, green line), (B) relative humidity in the exposure chamber and (C) gas flows (HCS: humidified carrier stream; VCS: vaporized carrier stream). Evaporated medium is shown as percentage of initial filing level of the individual wells of a 24-well plate of the exposure (upper D) and of the reference chamber (lower D) after 72 h of exposure.

S1-Table 9: Evaporated media (% of initial volume) in the exposure (A) and the reference chamber (B) in each individual well of a 24-well plate after 72 h of exposure (grey = wells with transwell inserts, white = medium filing only).

(A) Exposure chamber

Amount of evaporated medium

[% of initial volume]

D	C	B	A	
8.51	8.13	8.00	8.24	1
7.73	6.20	7.39	7.31	2
4.83	3.90	3.09	3.12	3
4.50	4.62	3.27	2.63	4
7.58	6.38	6.02	6.54	5
8.08	7.69	7.40	5.32	6

(B) Reference chamber

Amount of evaporated medium

[% of initial volume]

D	C	B	A	
7.73	6.54	7.15	7.91	1
6.19	5.35	5.08	5.93	2
3.08	3.73	2.65	2.35	3
1.92	2.54	2.31	2.54	4
5.50	5.00	5.31	5.27	5
7.73	7.31	6.77	7.46	6

S1-Fig.9: 0 ppm exposure, no.3

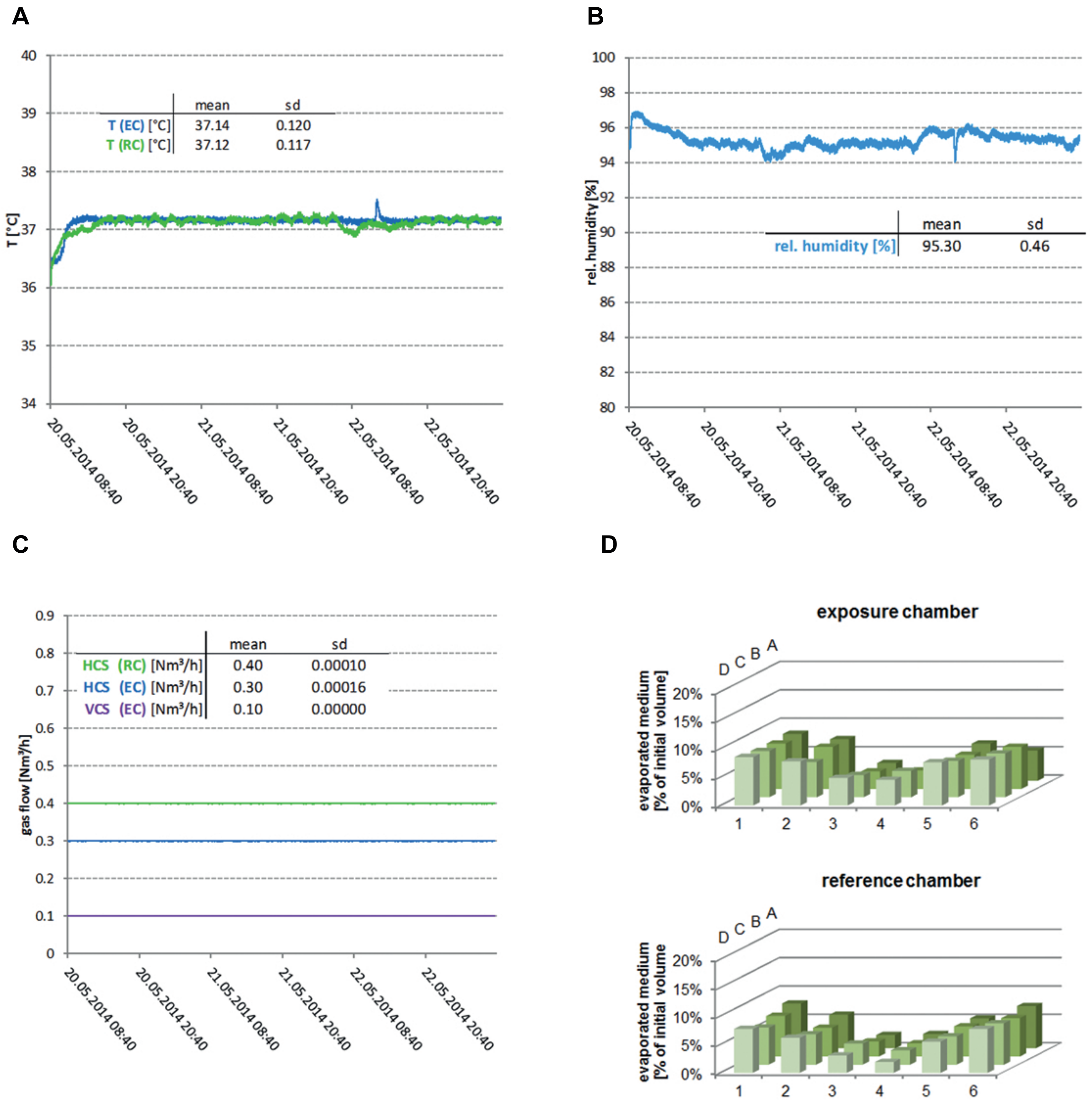

S1-Fig. 10: Calibration of the Aerolaser 4021 and control measurements. (A) For the calibration, the 5 volt signal is set to 0 V at zero level and to 4 V at 500 ppb formaldehyde (blue). The green line represents control measurement in the exposure chamber for which the exposure platform´s setpoint was adjusted at 500 ppb, showing the reliability of the dosing and exposure. (B) Comparison between the zero-level calibration (red) for which the zero air is supplied internally by the Aerolaser system by a very effective catalyst (potential formaldehyde contained in ambient air is held back in adsorbing material) and the baseline level of formaldehyde traces in the air fed into the platform (purple), which had an approximate formaldehyde concentration of 4.5 ppb.

S1-Fig.10

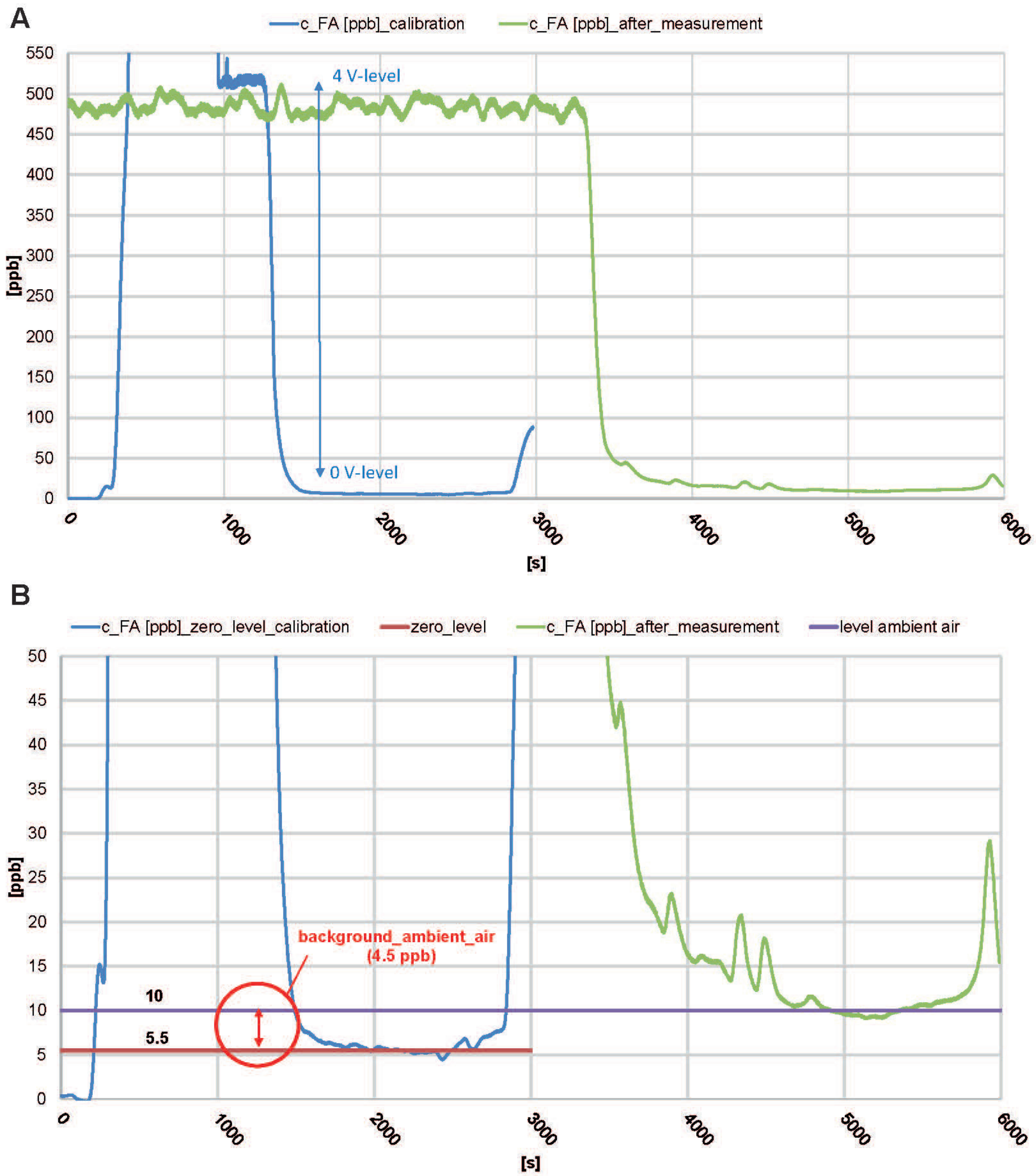

Supplemental file S2

Manuscript title

Cellular reactions to long-term volatile organic compound (VOC) exposures

Authors

Johanna M Gostner[1*], Johannes Zeisler[1,2,+], Mohammad Tauqeer Alam[3], Peter Gruber[1], Dietmar Fuchs[4], Kathrin Becker[4], Kerstin Neubert[5,++], Markus Kleinhappl[2], Stefan Martini[2], Florian Überall[1]

Affiliations

[1] Division of Medical Biochemistry, Biocenter, Medical University of Innsbruck, Austria

[2] Bioenergy 2020+, Graz, Austria

[3] Division of Biomedical Sciences, Warwick Medical School, University of Warwick, UK

[4] Division of Biological Chemistry, Biocenter, Medical University of Innsbruck, Austria

[5] ATLAS Biolabs GmbH, Berlin, Germany

+ current address: Energy and Environmental Technology Group, Fritz Egger GmbH & Co. OG, St. Johann in Tirol, Austria

++ current address: Department of Mathematics and Computer Science, Freie Universität Berlin, Germany

Supplemental file S2 – Gene expression

S2-Fig.1 Analysis of differentially expressed transcripts due to formaldehyde exposure. An overview of the comparative analysis of distinct treatments to obtain the differentially expressed gene sets can be found in the main manuscript, figure 4 A. Comparisons 1 to 3 (comp1, comp2 and comp3) refer to the cells that were contemporaneously cultivated in the reference chamber, while in comparisons 4 and 5 (comp4, comp5) the samples exposed to 0 ppm formaldehyde in the exposure chamber were used as control. This was done to exclude random background fluctuations due to chamber and time effect. Shown here are the Venn diagrams of the overlap of differentially expressed genes in each comparison using a *p*-value cutoff of < 0.05 (A) and using both a fold-change threshold of 2 and a *p*-value cutoff of < 0.05 (B). Numbers in bold are the transcripts that are common for 0,1 and 0.5 ppm treatment or individual for each of the exposures (e.g. for A: 18 – common, 312 – 0.1 ppm and 351 – 0.5 ppm exposure).

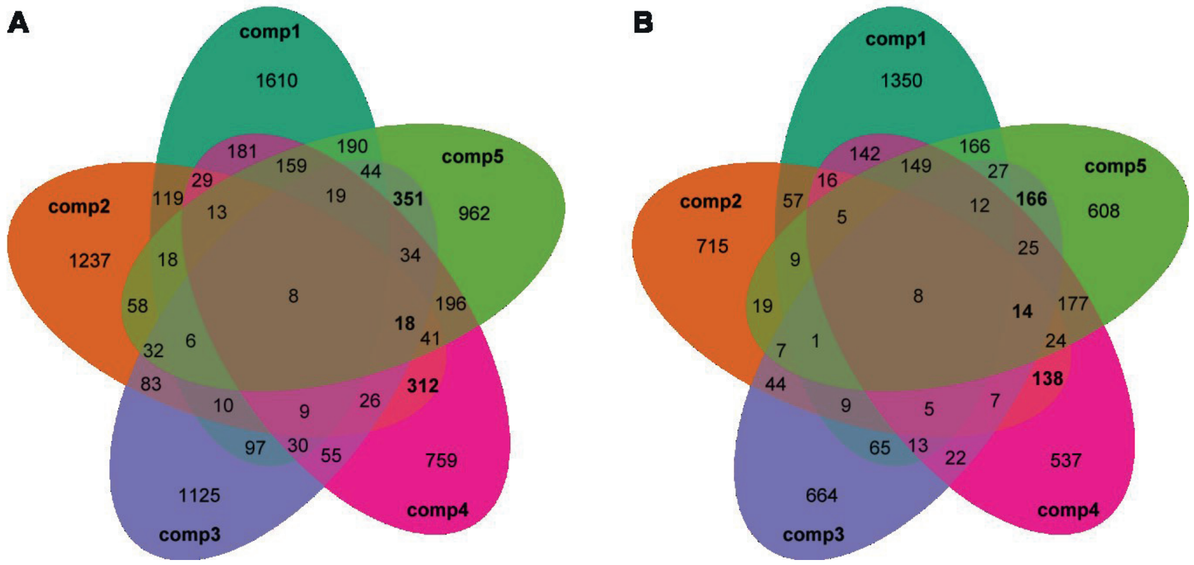

S2-Fig.2 Common genes – potential exposure markers. Additional statistical analysis of the potential exposure biomarkes indicated in the common transcript list in Table 3 of the main manuscripts. (A) Venn diagram showing the overlap of the common differentially expressed genes in 0.1 and 0.5 ppm exposure compared with controls in the ANOVA (blue) and the eBayes approach (green). Heatmaps showing the normalized expression of the resulting probesets using ANOVA/Tukey's HSD (B) and eBayes (C) approach. Transcripts marked with an asterisk are hits found with both methods. (RC = reference chamber, EC = exposure chamber).

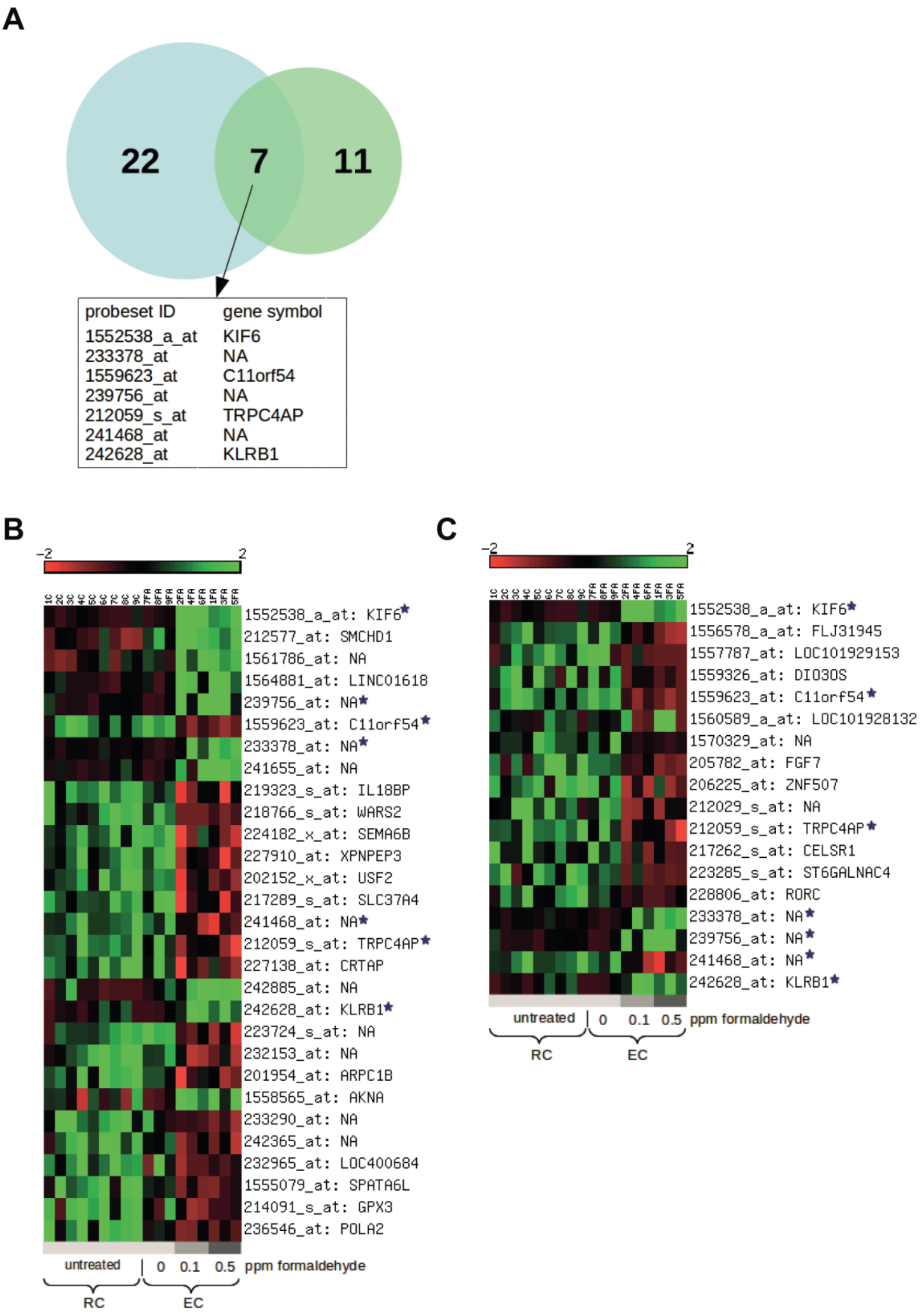

Supplement: Supplementary Information [file srep37842-s1.pdf]
